# Lectin-like oxidized low-density lipoprotein receptor 1 attenuates pneumonia-induced lung injury

**DOI:** 10.1172/jci.insight.149955

**Published:** 2022-12-08

**Authors:** Filiz T. Korkmaz, Anukul T. Shenoy, Elise M. Symer, Lillia A. Baird, Christine V. Odom, Emad I. Arafa, Ernest L. Dimbo, Elim Na, William Molina-Arocho, Matthew Brudner, Theodore J. Standiford, Jawahar L. Mehta, Tatsuya Sawamura, Matthew R. Jones, Joseph P. Mizgerd, Katrina E. Traber, Lee J. Quinton

**Affiliations:** 1 Division of Immunology and Infectious Disease, Department of Medicine, UMass Chan Medical School, Worcester, Massachusetts, USA.; 2 Pulmonary Center,; 3 Department of Microbiology, and; 4 Flow Cytometry Core Facility, Boston University School of Medicine, Boston, Massachusetts, USA.; 5 Division of Pulmonary and Critical Care Medicine, Department of Medicine, University of Michigan, Ann Arbor, Michigan, USA.; 6 Department of Internal Medicine, College of Medicine, University of Arkansas for Medical Sciences and the Central Arkansas Veterans Healthcare System, Little Rock, Arkansas, USA.; 7 Department of Molecular Pathophysiology, Shinshu University School of Medicine, Matsumoto, Nagano, Japan.; 8 Department of Medicine and; 9 Department of Biochemistry, Boston University School of Medicine, Boston, Massachusetts, USA.

**Keywords:** Immunology, Inflammation, Bacterial infections, Cellular immune response, Innate immunity

## Abstract

Identifying host factors that contribute to pneumonia incidence and severity are of utmost importance to guiding the development of more effective therapies. Lectin-like oxidized low-density lipoprotein receptor 1 (LOX-1, encoded by *OLR1*) is a scavenger receptor known to promote vascular injury and inflammation, but whether and how LOX-1 functions in the lung are unknown. Here, we provide evidence of substantial accumulation of LOX-1 in the lungs of patients with acute respiratory distress syndrome and in mice with pneumonia. Unlike previously described injurious contributions of LOX-1, we found that LOX-1 is uniquely protective in the pulmonary airspaces, limiting proteinaceous edema and inflammation. We also identified alveolar macrophages and recruited neutrophils as 2 prominent sites of LOX-1 expression in the lungs, whereby macrophages are capable of further induction during pneumonia and neutrophils exhibit a rapid, but heterogenous, elevation of LOX-1 in the infected lung. Blockade of LOX-1 led to dysregulated immune signaling in alveolar macrophages, marked by alterations in activation markers and a concomitant elevation of inflammatory gene networks. However, bone marrow chimeras also suggested a prominent role for neutrophils in LOX-1–mediated lung protection, further supported by LOX-1^+^ neutrophils exhibiting transcriptional changes consistent with reparative processes. Taken together, this work establishes LOX-1 as a tissue-protective factor in the lungs during pneumonia, possibly mediated by its influence on immune signaling in alveolar macrophages and LOX-1^+^ airspace neutrophils.

## Introduction

Pneumonia is a leading cause of death in the United States and is the most lethal infection-related disease worldwide (1, 2). Additionally, lower respiratory infections are a significant source of morbidity, increasing the risk of subsequent cardiovascular disease, neurocognitive dysfunction, and recurrent infections (3, 4). The severity of pneumonia outcomes is exceptionally heterogenous, ranging from mild to severe or even fatal as a function of age, immune status, and the presence of preexisting conditions (5), such as, for example, those highlighted in the COVID-19 pandemic (6). Treatment for patients with pneumonia remains overly reliant on supportive care and antibiotics and is further complicated by extremely diverse etiology (3, 7), limiting the potential benefit of microbe-specific therapies. Ultimately, overcoming these challenges will demand a better understanding of host pathways controlling pneumonia susceptibility, thus possibly leading to the development of novel clinical interventions in individuals with or at risk for respiratory infections (8).

Whether or not lung infections progress to pneumonia largely depends on integrated contributions of host systems that reduce pathogen burden and limit tissue injury (9–11). Failure to achieve the latter can result in acute lung injury and its most severe form, acute respiratory distress syndrome (ARDS). As a potential determinant of tissue homeostasis in pneumonic airspaces, we have found that the lectin-like oxidized low-density lipoprotein receptor 1 (LOX-1, encoded by *OLR1*) plays multifunctional roles during infection. LOX-1 is a class E scavenger receptor, expressed on a number of cell types, including but not limited to macrophages, endothelial cells, smooth muscle cells, and myeloid-derived suppressor cells (12, 13). Like most scavenger receptors, it is promiscuous and binds to a wide range of ligands (14), including its canonical ligand oxidized low-density lipoprotein (oxLDL), a common byproduct of oxidative stress. In the vasculature, LOX-1 is well recognized to promote the development of atherosclerosis through oxLDL uptake, cell death, inflammatory cytokine production, and the recruitment and development of foam cells within atherosclerotic plaques (13). Yet, whether and how this receptor functions to shape inflammatory responses within the airspaces of the lung is unknown.

Initial observations of substantial pneumonia-induced LOX-1 expression prompted us to determine its source of production, expression dynamics, and biological function in the context of respiratory infection. Surprisingly, our findings implicate the airspaces of the lung as a unique niche for LOX-1–driven tissue protection, possibly through regulation of alveolar macrophage and recruited neutrophil activity, in order to maintain tissue homeostasis in the wake of infection. To our knowledge, this is the first report to examine the biology of LOX-1 in the alveolar compartment and its influence on pneumonia outcomes.

## Results

### LOX-1 and its major ligands accumulate in the lungs during pneumonia.

**Figure 1:**
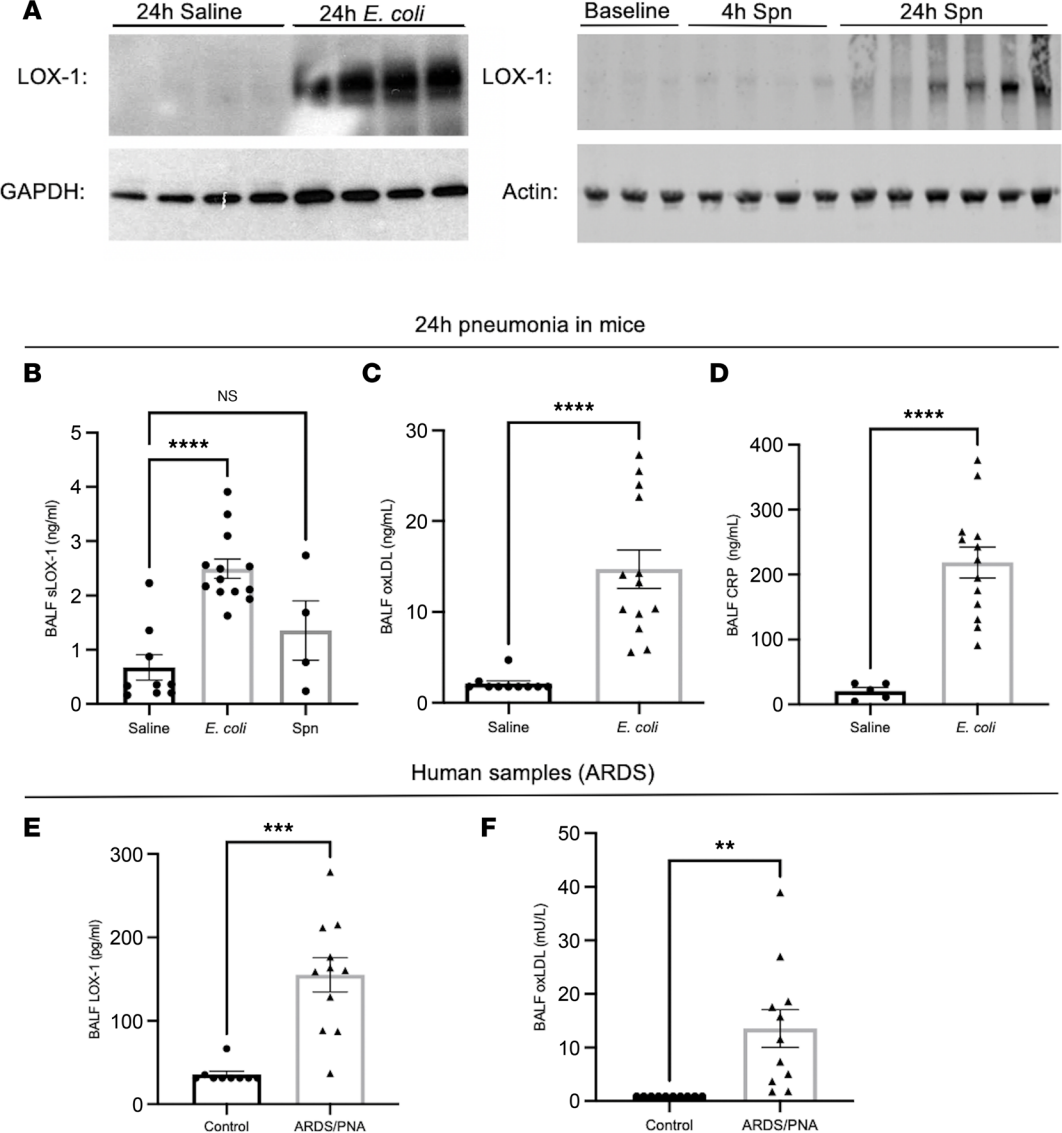
LOX-1 and its ligands are induced during pneumonia in mouse and human lungs. Age-matched C57BL/6 mice (*n* = 4–13 per group) were intratracheally instilled with (**A**–**D**) *E*. *coli* or (**A** and **B**) *S*. *pneumoniae*. LOX-1 protein was subsequently measured in (**A**) whole lung homogenate and (**B**) bronchoalveolar lavage fluid (BALF). In *E*. *coli*–infected mice, LOX-1 ligands (**C**) oxLDL and (**D**) CRP were measured in BALF. Finally, BALF was obtained from human patients with ARDS as a result of a confirmed pneumonia diagnosis (PNA) (*n* =11) and non-ARDS healthy controls (*n* = 9), and (**E**) LOX-1 protein and (**F**) oxLDL were measured. Mouse data are represented as mean ± SEM with individual data points representative of mice from 2–3 independent experiments. Human data are similarly represented as mean ± SEM with individual data points representative of different patients. ****P < 0.0001, ***P < 0.001, **P < 0.01 for 1-way ANOVA with Dunnett’s post hoc test (**B**) or 2-tailed Welch’s *t* test (**C**–**F**).

To address whether LOX-1 content is dynamically regulated in response to respiratory infection, we induced left lobar pneumonias in adult C57BL/6 mice through intratracheal (i.t.) instillations of *Escherichia coli* (*E*. *coli*) and *Streptococcus pneumoniae* (serotype 3) (*S*. *pneumoniae*). These 2 pathogens were chosen to represent prominent nosocomial and community-acquired pneumonia-causing microorganisms (3). Measurement of LOX-1 immunoreactivity in pneumonic left lung lobes revealed a marked induction of LOX-1 during acute infection (Figure 1A). In addition to the membrane-bound form of LOX-1, numerous reports have shown that LOX-1 can be cleaved by several proteases (15), yielding a soluble form (sLOX-1) that serves as a reliable biomarker for vascular injury (16). As shown in Figure 1B, airspace concentrations of sLOX-1 (as determined in bronchoalveolar lavage fluid, BALF) were significantly elevated in response to *E*. *coli*, directly correlating with its accumulation in lung homogenates, with similar trends observed following challenges with *S*. *pneumoniae* (Figure 1B).

LOX-1 ligands are diverse, with the most canonical and widely recognized as oxLDL. To address the potential for LOX-1 ligands to engage this receptor, we measured oxLDL concentrations in the BALF of pneumonic mice and observed substantial accumulation (Figure 1C), with similar results observed for C-reactive protein (CRP), an acute phase protein that also functions as a LOX-1 ligand (14) (Figure 1D). To address whether these findings extend to pneumonia in humans, we measured sLOX-1 and oxLDL in BALF obtained from patients with pneumonia-induced ARDS (abbreviated as ARDS/PNA) and compared levels with those observed in BALF specimens from healthy volunteers. In agreement with our murine findings, sLOX-1 and oxLDL were significantly increased in human BALF (Figure 1, E and F). This finding was reproducible in BALF specimens from ARDS patients without a clinical diagnosis of pneumonia (Supplemental Figure 1; supplemental material available online with this article; https://doi.org/10.1172/jci.insight.149955DS1; full immunoblot images available in online supplemental material), implicating the involvement of intrapulmonary LOX-1 activity in settings of lung injury beyond that elicited by acute lower respiratory infection. These data indicate that LOX-1 and its ligands are elevated in the lungs because of infection, where they may contribute to immunity and/or immunopathology.

### Global LOX-1 deficiency increases neutrophil recruitment and antibacterial defense, suggesting antiinflammatory roles during pneumonia.

Total LOX-1 deletion has been shown to reduce lung injury in response to septicemia (17), as well as vascular inflammation in mouse models of myocardial reperfusion injury and atherosclerosis (18, 19). In fact, prior studies of LOX-1 almost uniformly support a proinflammatory and injurious role for this receptor, albeit in diverse settings (13, 14). Given the substantial increase in lung LOX-1 during pneumonia, we sought to determine whether LOX-1 deficiency would blunt the inflammatory consequences of lung infection, possibly influencing antimicrobial defense. To do so, wild-type and LOX-1–knockout mice were intratracheally infected with *E*. *coli* or *S*. *pneumoniae* for 24–30 hours in order to determine the influence of LOX-1 on pulmonary edema, neutrophilic inflammation, and bacterial clearance. While no differences were detected between knockout (KO) and wild-type (WT) mice in measurements of alveolar edema (total protein; Supplemental Figure 2, A, D, and F) in pneumonic BALF, neutrophil recruitment was significantly *elevated* in *E*. *coli*–challenged mice devoid of LOX-1 and was unchanged during pneumococcus infections (Supplemental Figure 2, B, E, and G). Collectively, these data indicate that endogenous LOX-1 limits neutrophilic inflammation, which is enhanced in KO mice in association with improved bacterial clearance (Supplemental Figure 2C). Although genetic LOX-1 deficiency was not sufficient to promote alveolar edema, these findings suggest that if anything, LOX-1 has a dampening effect on local immune signaling in the context of pneumonia, contrasting previous reports in other models of inflammatory injury (17, 19).

### Intratracheal inhibition of LOX-1 exacerbates pneumonia-induced injury.

**Figure 2:**
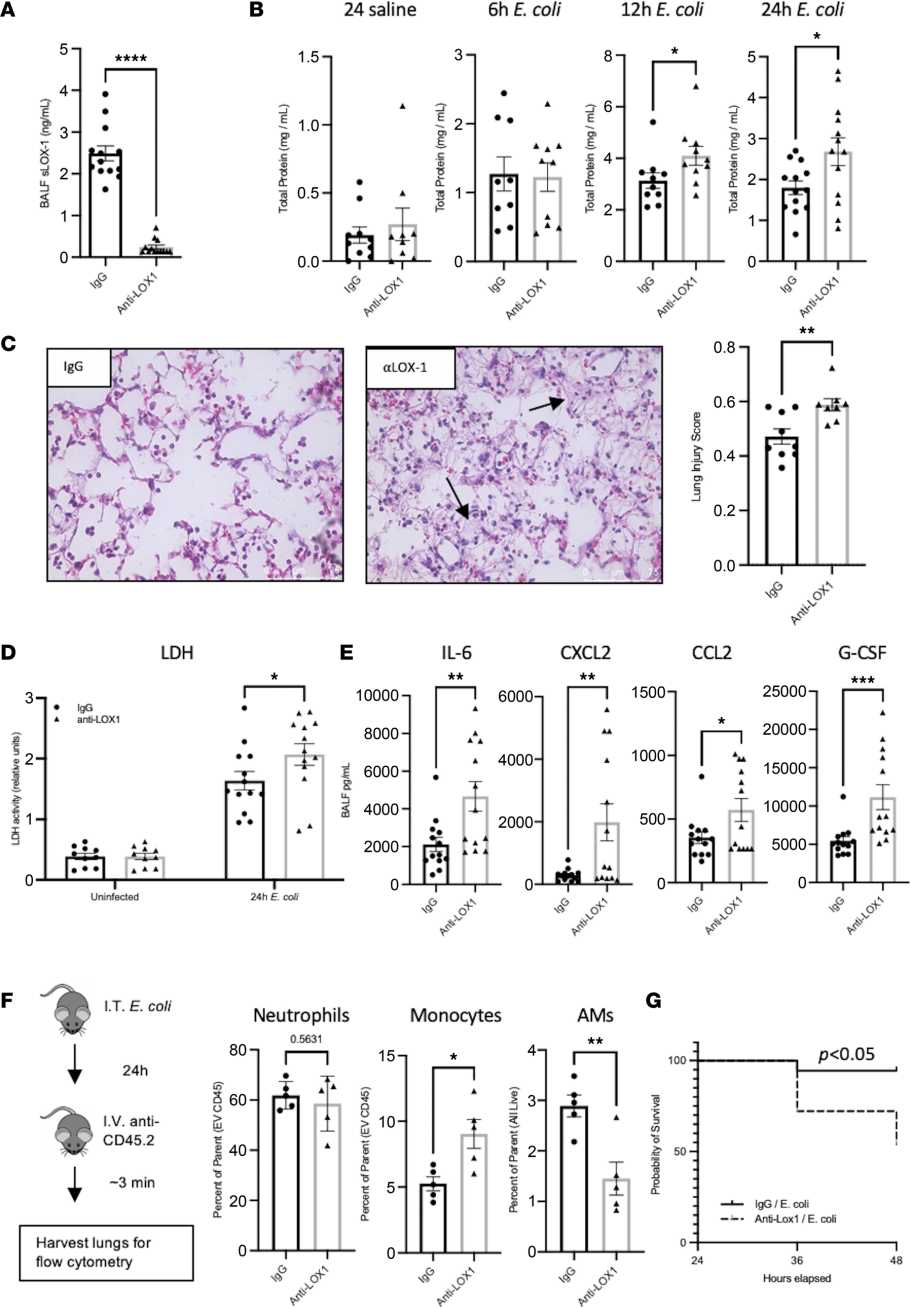
Local inhibition of LOX-1 exacerbates lung injury and inflammation during pneumonia. Age-matched C57BL/6 mice were intratracheally treated with 10 μg anti–LOX-1 or control IgG and *E*. *coli* (*n* = 5–13 per group depending on experimental outcome). (**A**) Neutralization of LOX-1 was confirmed by measuring BALF sLOX-1 at 24 hours after anti–LOX-1 or IgG treatment and infection with *E*. *coli*. (**B**) Total protein was measured in BALF at the indicated time points as an index of alveolar edema. (**C**) H&E staining was performed on paraffin-embedded lung sections collected from anti–LOX-1– and IgG-treated mice at 24 hours postinfection. Original magnification, ×40. Arrows point to examples of elevated injury, with visual evidence of proteinaceous edema and fibrin deposition. (**D**) Lactate dehydrogenase (LDH) levels were measured in BALF collected from mice with or without infection in the presence of anti–LOX-1 or IgG treatment. (**E**) Cytokine concentrations were measured in BALF at 24 hours postinfection. (**F**) Flow cytometry was performed on collagenase-digested lungs collected from anti–LOX-1– and IgG-treated mice infected with *E*. *coli* for 24 hours and intravenously treated with a fluorescently labeled anti-CD45.2 Ab 3 minutes prior to euthanasia to determine extravascular neutrophil (i.v. CD45^–^CD45^+^CD11b^+^Ly6G^+^), inflammatory monocyte (i.v. CD45^–^CD45^+^CD11b^+^Ly6C^hi^), and alveolar macrophage (i.v. CD45^–^CD45^+^CD11c^+^SiglecF^+^) numbers. (**G**) Survival was determined through 48 hours. Data are represented as mean ± SEM with individual data points representative of mice from 2–3 independent experiments. ****P < 0.0001, ***P < 0.001, **P < 0.01, *P < 0.05 for 2-tailed Welch’s *t* test (**A**), Mann-Whitney test (**B** [12 hours], **D** [G-CSF]), 2-tailed, unpaired *t* test (**B** [24 hours], **C** and **D** [except G-CSF], **F**), or log-rank (Mantel-Cox) test (**G**). EV, extravascular.

Results obtained using the whole-animal model of LOX-1 deficiency described above are insufficient to resolve whether or how LOX-1 functions specifically within the pulmonary airspaces. Given this limitation, we employed a pharmacological approach to achieve local LOX-1 inhibition through i.t. instillation of a neutralizing LOX-1 Ab. This approach has been successfully used to block the biological function of LOX-1 (20), and as we have previously shown, i.t. administration of neutralizing Abs is highly effective at consolidating neutralization to the pulmonary airspaces (10). To do so, we intratracheally co-instilled 10 μg of neutralizing anti–LOX-1 IgG or a nonspecific IgG control with *E*. *coli* or *S*. *pneumoniae*. As shown in Figure 2A, instillation of neutralizing Ab completely abolished detection of sLOX-1 in pneumonic BALF from *E*. *coli*–infected mice, with similar results seen during *S*. *pneumoniae* infection (data not shown), supporting highly effective functional blockade.

In contrast with the existing paradigm of LOX-1–driven injury and in support of antiinflammatory roles for LOX-1 as observed in the LOX-1–KO mice (Supplemental Figure 2), local blockade of this receptor exacerbated injury, as evidenced by elevated proteinaceous edema (total protein in the BALF) following infection with *E*. *coli* but not control (anti–LOX-1/saline) mice (Figure 2B). Elevated injury occurred as early as 12 hours postinfection in mice treated with anti–LOX-1 and endured through at least 24 hours (Figure 2B). Total protein was similarly elevated, albeit nonsignificantly, in *S*. *pneumoniae–*infected and LPS-treated mice (Supplemental Figure 3), which does not elicit the overall degree of lung injury observed following an *E*. *coli* infection. Elevations in proteinaceous edema were further corroborated by histopathological evidence of increased injury in anti–LOX-1–treated pneumonic mice (Figure 2C), significantly greater lung injury score as determined using established guidelines (Figure 2C) (21), and increased lung cell death as measured by BALF lactate dehydrogenase (LDH) activity (Figure 2D). Consistent with increased injury, multiple inflammatory cytokines were significantly upregulated following LOX-1 neutralization in response to pneumonias induced by either *E*. *coli* or *S*. *pneumoniae* (Figure 2E and Supplemental Figure 4B), some of which were the same across both challenges (CXCL2, G-CSF). Moreover, some of these cytokines were upregulated at 2 different time points (12 hours and 30 hours) following LOX-1 neutralization during pneumonia, but not at early time points (6 hours) postinfection (Supplemental Figure 4A), which is consistent with the onset of injury no earlier than 12 hours (Figure 2B). Unlike results observed in LOX-1–KO mice, however, neutrophil accumulation was not elevated in anti–LOX-1–treated mice challenged with either *E*. *coli* or *S*. *pneumoniae* (Figure 2F and Supplemental Figure 5), likely owing to the airspace-confined nature of the Ab approach. In fact, BALF neutrophil counts were diminished following LOX-1 blockade at 12 hours postinfection despite increased edema, strongly suggesting that the latter is unrelated to the former (Supplemental Figure 5A).

As an alternative approach, we distinguished extravascular neutrophils, inflammatory monocytes, and alveolar macrophages in single-cell lung suspensions, which were discriminated by intravital staining of circulating hematopoietic cells (α-CD45.2) prior to euthanasia. This approach revealed a significant increase in the fraction of lung-recruited monocytes (Figure 2F), concomitant with reduced alveolar macrophages, which is consistent with the observed increase in the monocyte-recruiting cytokine, CCL2 (Figure 2E). As with BALF neutrophils, however, extravascular lung neutrophils measured in lung digest specimens were unaffected by anti–LOX-1 treatment. Importantly, bacterial burdens were also unaffected by local LOX-1 blockade (Supplemental Figure 5), indicating that elevated injury was unlikely secondary to alterations in pathogen burden. Finally, longer experiments were initiated to determine the extent to which anti–LOX-1–treated mice recover from infection compared with IgG controls, but these studies were terminated due to mortality in the anti–LOX-1 group observed by as early as 36 hours (Figure 2G). Overall, these data suggest that LOX-1 in the airspaces is protective, limiting lung injury and immunopathology.

### Systemic blockade of LOX-1 is insufficient to alter pneumonia outcome.

Intravenous administration of anti-LOX-1 Ab has been widely used to target LOX-1, leading to reductions in inflammation, oxidative stress, and leukocyte infiltration in response to endotoxin-mediated lung injury, diabetic nephropathy, and vascular injury (22–24). Moreover, LOX-1 inhibitors, including anti–LOX-1 Ab therapies, are currently being considered for clinical use in cardiovascular disease (25). To determine the effects of systemic LOX-1 blockade during pneumonia, we treated mice intravenously with 10 μg of neutralizing anti–LOX-1 IgG or a nonspecific IgG control prior to intratracheal infection with *E*. *coli*. While intravenous anti–LOX-1 administration was sufficient to reduce BALF sLOX-1 accumulation, likely due to Ab extravasation by the 24-hours time point (Supplemental Figure 6A), no differences were detected in lung injury or inflammatory cytokines (Supplemental Figure 6, B and C), which, if anything, trended lower in anti–LOX-1–treated mice (similar to prior studies and opposite of that illustrated in Figure 2). Taken together, intrapulmonary but not systemic inhibition of LOX-1 enhances inflammatory injury, which contrasts the existing precedent for LOX-1–mediated damage in the vasculature and elsewhere (13). These unanticipated findings implicate the airspaces as a niche for LOX-1–mediated protection.

### LOX-1 is transcriptionally dynamic in nonhematopoietic lung cells but unchanged on their surface.

**Figure 3:**
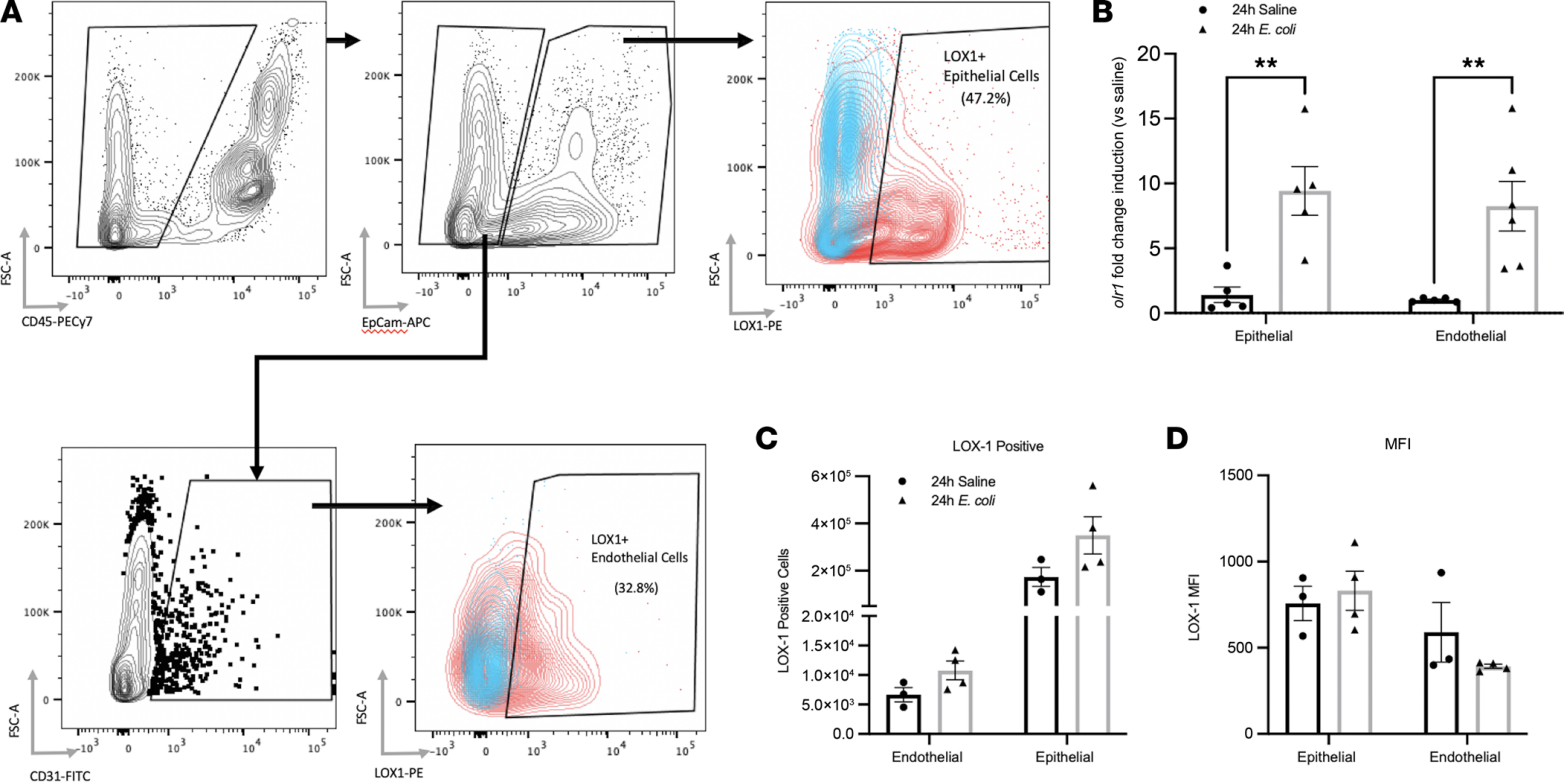
Expression of LOX-1 is induced in epithelial and endothelial cells during pneumonia with no change in surface expression. To identify the cellular sources of LOX-1, (**A**) epithelial (7AAD^–^CD45^–^EpCAM^+^) and endothelial (7AAD^–^CD45^–^EpCAM^–^CD31^+^) cells were isolated by FACS following either elastase (mRNA expression) or dispase (surface expression) digestion of lungs collected from age-matched C57BL/6 mice (*n* = 3–5 per group) intratracheally treated with saline or *E*. *coli* for 24 hours. (**B**) *Olr1* gene expression was determined by RT-qPCR. Sorted cells were further gated into LOX-1^+^ and LOX-1^–^ by flow cytometry, and (**C**) total LOX-1^+^ cells and (**D**) LOX-1 median fluorescence intensity (MFI) were determined. Data are represented as mean ± SEM with individual data points representative of mice from 1–2 independent experiments. **P < 0.01 for 2-way ANOVA with Holm-Šídák post hoc test.

Expression of LOX-1 has been reported on numerous cell types, including but not limited to endothelial cells, macrophages, smooth muscle cells, and DCs (13, 26). To first assess the expression of LOX-1 on nonhematopoietic cells in the lung, mice were i.t. treated with either *E*. *coli* or saline for 24 hours, and endothelial (CD45^–^EpCAM^–^CD31^+^) and epithelial (CD45^–^EpCAM^+^) cells were isolated by enzymatic digestion and cell sorting (Figure 3A). These cell types were selected due to their established importance in maintaining lung tissue integrity (27, 28), along with known effects of LOX-1 itself, particularly in the case of endothelium (29). Significant induction of LOX-1 mRNA (*Olr1*) was revealed by real-time quantitative PCR (RT-qPCR) in both cell types following 24 hours of pneumonia (Figure 3B). Flow cytometry results indicated that a large fraction of epithelial (35.1% ± 7.9%) and endothelial (54.5% ± 12.6%) cells had detectable levels of surface LOX-1; however, neither the number of LOX-1^+^ cells (Figure 3C) nor its surface density (Figure 3D) was impacted by infection. Surface staining was repeated using alternative digestion methods (collagenase and elastase) to determine whether the discordance between mRNA and surface expression was due to enzymatic cleavage of the receptor, but the results were identical across all digestion methods (data not shown). Therefore, while these cell types have the capacity to respond to LOX-1 ligands in other settings, our own findings suggest that they are unlikely contributors to the large increase in total lung LOX-1 elicited by infection (Figure 1).

### Alveolar macrophages are a prominent and inducible source of lung LOX-1.

**Figure 4:**
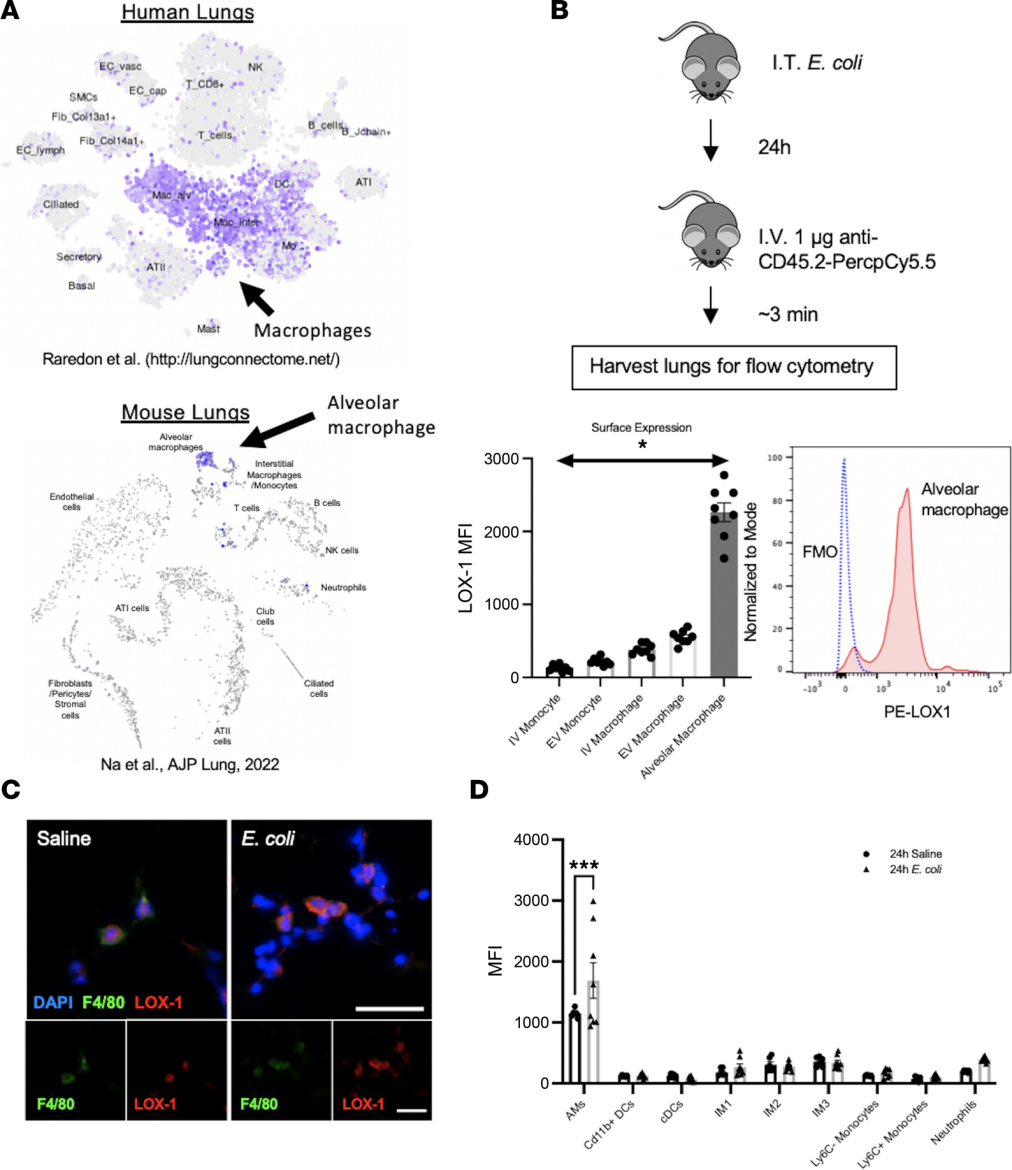
Airspace macrophages exhibit abundant LOX-1 expression that is further induced with infection. (**A**) To identify which lung cells exhibit the highest expression of LOX-1, we utilized a publicly available single-cell RNA-sequencing data set (31) and our own data set (30) from human and mouse lungs, respectively, to visualize cell-specific *Olr1* gene expression (highlighted in purple). (**B**) To assess recruited monocyte versus resident macrophage LOX-1 expression, age-matched C57BL/6 mice (*n* = 8) were intratracheally infected with *E*. *coli* for 24 hours, and anti-CD45.2 Ab was administered intravenously 3 minutes prior to euthanasia. Flow cytometry was used to measure intra- and extravascular LOX-1 expression on monocytes and macrophages. Alveolar macrophage expression (red histogram) was compared with fluorescence minus one control (FMO; blue dotted line). (**C**) Immunofluorescence of LOX-1 (red) versus F4/80 (green) was performed on frozen lung sections from mice intratracheally instilled with saline or *E*. *coli* for 24 hours (*n* = 3 per group) (scale bar = 25 μm). (**D**) To comprehensively assess myeloid LOX-1 expression, flow cytometry was performed on collagenase-digested lungs collected from C57BL/6 mice treated with either saline or *E*. *coli* for 24 hours (*n* = 6–8 per group). Data are represented as mean ± SEM with individual data points representative of mice from 2 independent experiments. ***P < 0.001, *P < 0.05 by 1- or 2-way ANOVA and Holm-Šídák post hoc test.

The lack of LOX-1 surface induction on epithelial and endothelial cells prompted us to explore other cell types given the substantive increase in total lung LOX-1 upon infection (Figure 1). To comprehensively interrogate cell-specific LOX-1 expression, we leveraged a publicly available single-cell sequencing (scSeq) data set originating from Raredon et al. (http://lungconnectome.net/), and our own recently published scSeq data (30, 31), to determine LOX-1–expressing cells in the lungs of both mice and humans. The original study by Raredon et al. (31) was performed on all cells obtained from normal (donor) human lungs from 14 individuals, both male and female, ranging in age from 21 to 88 years old. While *Olr1* mRNA was sparsely expressed among several cell types, including endothelial and alveolar epithelial cells, it was dramatically enriched within the cluster identified as alveolar and interstitial macrophages (Figure 4A). We observed very similar results when analyzing data from our own recent study (30), where we collected cells from mice treated intratracheally with saline or *E*. *coli* in combination with control IgG as in our current work (Figure 4A). Based on this finding along with prior evidence that LOX-1 on macrophages can contribute to vascular injury (13), we assessed LOX-1 surface expression on intra- and extravascular monocytes (CD45^+^F4/80^–^CD11b^+^Ly6C^+^), interstitial (nonalveolar) macrophages (CD45^+^F4/80^+^CD11b^+^SiglecF^–^Ly6C^–^), and alveolar macrophages (CD45^+^F4/80^+^SiglecF^+^) collected from collagenase-digested lungs following 24 hours of pneumonia (Figure 4B). Intra- and extravascular cells were discriminated by intravital staining of circulating hematopoietic cells (α-CD45.2) prior to euthanasia and staining for all CD45-expressing cells (pan α-CD45). Interestingly, the intensity of LOX-1 surface expression was far greater on alveolar macrophages in comparison with all other monocytes and macrophages, irrespective of their location (Figure 4B). In fact, nearly 100% of alveolar macrophages expressed LOX-1 on their surface. Moreover, immunofluorescence staining for LOX-1 (red) with a macrophage cell marker (F4/80; green) revealed substantial overlap between LOX-1 and F4/80 in both saline-treated and *E*. *coli–*infected lungs (Figure 4C).

To specifically determine whether alveolar macrophage LOX-1 expression is altered by pneumonia and the degree to which this compares to other lung leukocytes, we generated lung single-cell suspensions from mice treated with saline or *E*. *coli* for 24 hours and employed the myeloid flow cytometry panel shown in Supplemental Figure 7. This strategy was sufficient to determine LOX-1 expression on alveolar macrophages (CD11b^–^CD64^+^CD11c^+^SiglecF^+^); interstitial macrophages subtypes 1 (CD11b^+^CD64^+^CD11c^–^SiglecF^–^MHCII^–^), 2 (CD11b^+^CD64^+^CD11c^–^SiglecF^–^MHCII^+^), and 3 (CD11b^+^CD64^+^CD11c^+^SiglecF^–^MHCII^+^) (32); CD11b^+^ dendritic cells (CD11b^+^CD64^–^CD11c^+^); classic dendritic cells (CD11b^–^CD64^–^CD11c^+^MHCII^+^); inflammatory monocytes (CD11b^+^CD64^–^CD11c^–^Ly6C^+^); patrolling monocytes (CD11b^+^CD64^–^CD11c^–^Ly6C^–^); and neutrophils (CD11b^+^Ly6G^+^). While the majority of macrophages expressed detectable LOX-1 (Figure 4B), alveolar macrophages also had strikingly elevated surface expression in comparison with other cell types (Figure 4D). Moreover, they were the only cell type analyzed in which LOX-1 surface expression was induced further upon infection (Figure 4D).

Next, to determine the proportion of inducible LOX-1 in whole lung homogenates (Figure 1A) originating from alveolar macrophages, they were depleted using clodronate-encapsulated liposomes administered intratracheally 72 hours before infection with *E*. *coli*. We and others have established this strategy as an effective way to reduce alveolar macrophage numbers (11, 33). We did not detect differences in LOX-1 expression using this approach (Supplemental Figure 8), suggesting that cells other than alveolar macrophages also contribute to pneumonia-induced elevations of lung LOX-1 expression. Notably, this result may be attributable to inherent limitations of this strategy, including but not limited to incomplete depletion, impaired defense, and/or altered neutrophil counts, the latter of which may contribute to the inducible LOX-1 pool (see below). Regardless, our combined results reveal alveolar macrophages as a potential candidate for LOX-1–mediated tissue protection based on the exquisitely high baseline expression, further induction with infection, and established roles for this cell type in promoting tissue repair following lung injury (34).

### Alveolar macrophages exhibit a dysregulated phenotype in the absence of LOX-1.

**Figure 5:**
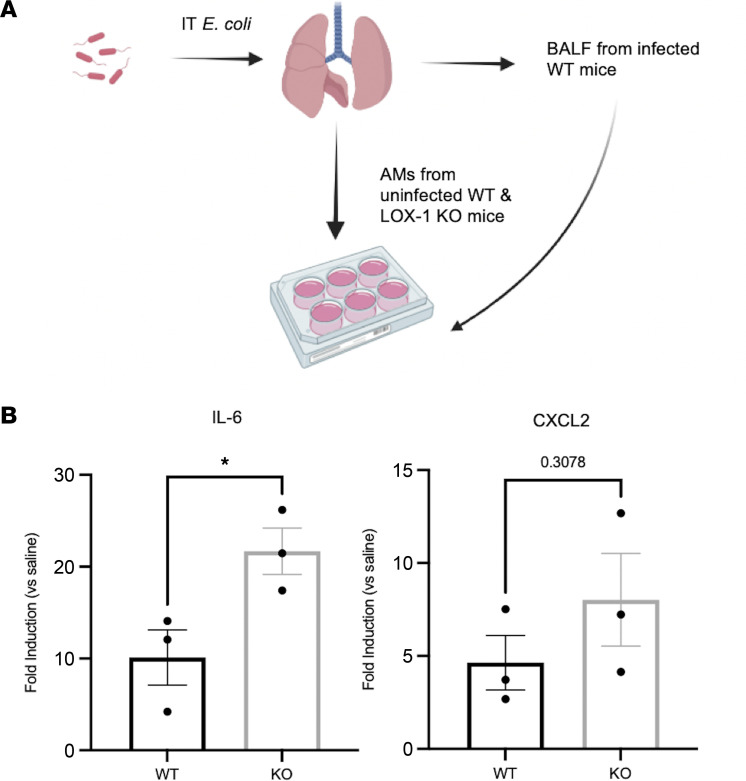
LOX-1–deficient alveolar macrophages exhibit an exaggerated cytokine response when stimulated with pneumonic airspace constituents. (**A**) Alveolar macrophages from age-matched C57BL/6 or LOX-1^–/–^ mice were isolated by bronchoalveolar lavage and stimulated ex vivo for 4 hours with cell-free BALF from WT mice infected with *E*. *coli* for 24 hours. (**B**) Gene expression for IL-6 and CXCL2 was measured by RT-qPCR. Data are represented as mean ± SEM with individual data points representative of data averages collected from 3 independent experiments. *P < 0.05 by unpaired, 2-tailed *t* test.

**Figure 6:**
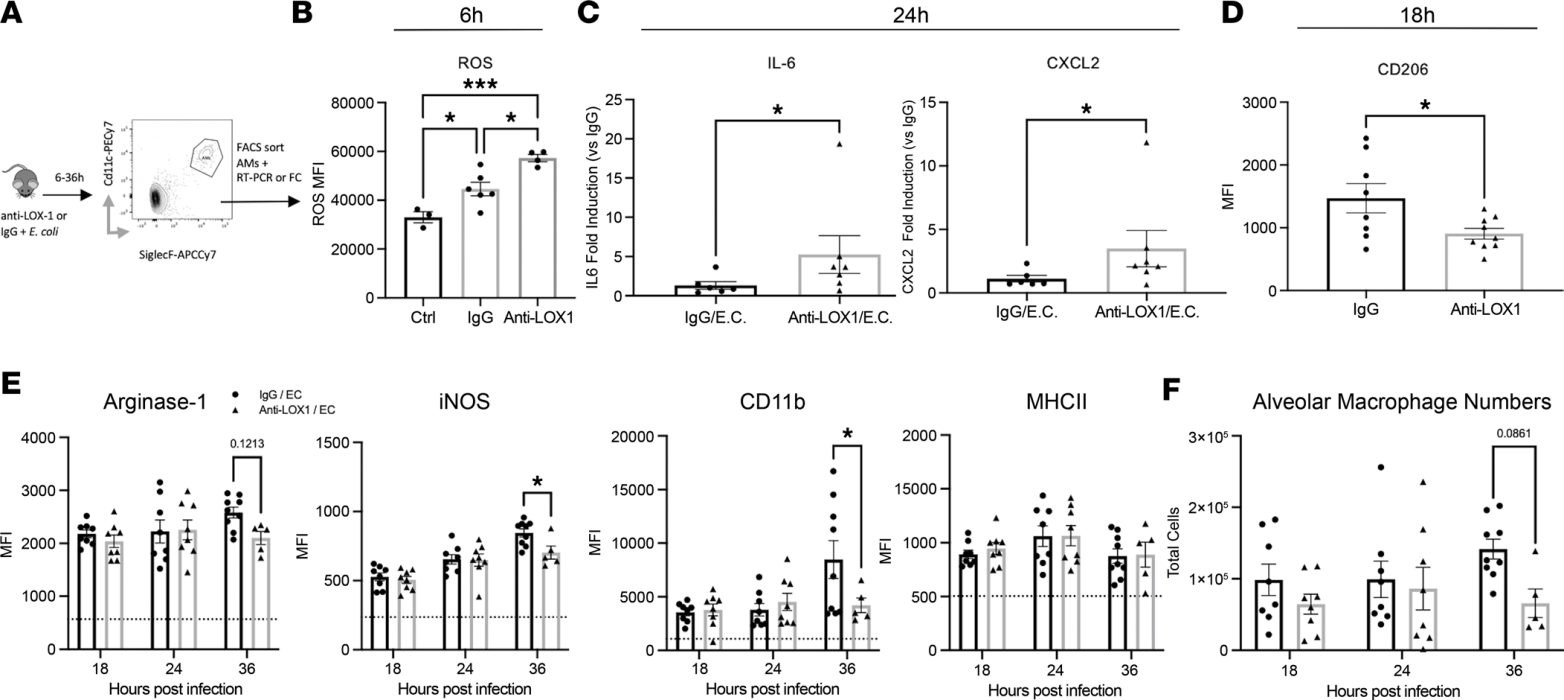
Alveolar macrophages exhibit a dysregulated phenotype with LOX-1 inhibition. (**A**) Age-matched C57BL/6 mice were intratracheally treated with 10 μg anti–LOX-1 IgG or control IgG and *E*. *coli* for 6–36 hours (*n* = 6–8 per group). FACS-sorted alveolar macrophages were subsequently analyzed by flow cytometry for (**B**) levels of reactive oxygen species (ROS) at 6 hours, (**C**) IL-6 and CXCL2 expression at 24 hours, or (**D**) CD206 expression at 18 hours after anti–LOX-1 or IgG treatment and infection. Intracellular flow cytometry was performed on fixed cells at 18–36 hours after anti–LOX-1 or control IgG treatment and *E*. *coli* infection to determine (**E**) Arginase 1, iNOS, MHCII, and CD11b expression (MFI) and (**F**) total alveolar macrophage numbers. Data are represented as mean ± SEM with individual data points representative of mice from 2–3 independent experiments. Statistical analysis was performed on normalized (log-transformed) IL-6 and CXCL2 gene expression (fold change). ***P < 0.001, *P < 0.05 for 1-way ANOVA with Holm-Šídák post hoc test (**B**); 2-tailed Welch’s *t* test (**C**); 2-tailed, unpaired *t* test (**D**); or 2-way ANOVA with Holm-Šídák post hoc test (**D** and **E**).

Based on the findings above, we wanted to specifically address how alveolar macrophages were affected by LOX-1 deletion or neutralization. As such, we isolated alveolar macrophages from WT and LOX-1^–/–^ mice and stimulated them with cell-free lavage fluid collected from WT mice 24 hours after *E*. *coli–*induced pneumonia. Here, we measured a significant increase in mRNA expression of IL-6 and a trend toward elevated CXCL2 in LOX-1^–/–^ macrophages (Figure 5), indicating an intrinsic role for LOX-1 in regulating macrophage responsiveness that does not require changes in the surrounding milieu. Next, we sorted alveolar macrophages from BALF of mice treated with anti-LOX-1 and IgG that were infected with *E*. *coli* for 6–36 hours (Figure 6A). Interestingly, macrophages collected from mice treated with anti–LOX-1 at 6 hours postinfection, a time preceding changes in cytokines or injury (Figure 2), exhibited increased levels of reactive oxygen species (ROS) (Figure 6B). Similar to ex vivo studies detailed above, both IL-6 and CXCL2 mRNA expression was significantly enhanced in anti–LOX-1 mice following 24 hours of pneumonia (Figure 6C). Both of these prominent proinflammatory cytokines/chemokines were also elevated in BALF of anti–LOX-1–treated mice infected with *E*. *coli* and *S*. *pneumoniae* (Figure 2E and Supplemental Figure 4A), suggesting alveolar macrophages are directly or indirectly contributing to enhanced inflammation with LOX-1 inhibition.

Macrophages are known for their phenotypic heterogeneity and sensitivity to alterations in tissue microenvironments. Expression of proinflammatory (inducible NO synthase [iNOS], MHCII, CD80/86) and tissue-resolving (CD206, Ym1, Arginase 1, CD163) markers is routinely used to classify macrophage phenotypes (35). Based on enhanced cytokine expression of alveolar macrophages isolated from anti–LOX-1–treated BALF, we subsequently measured CD206 surface expression by flow cytometry as a proxy of M2-like macrophage phenotype. Consistent with increased injury, CD206 surface expression was significantly lower in anti–LOX-1–treated mice at 18 hours postinfection (Figure 6D), suggesting LOX-1 may normally promote M2-like polarization and tissue resolution. Using intracellular flow cytometry, we then measured a subset of phenotypic markers (Arginase 1, iNOS, MHCII, and CD163), which classically represent pro- and antiinflammatory function following LOX-1 inhibition at 18–36 hours of *E*. *coli* infection. Curiously, macrophages that were recovered displayed an altered expression pattern of the phenotypic markers arginase 1 and iNOS. Arginase 1, which is typically lower in M1-like, inflammatory macrophages, was reduced by 36 hours in alveolar macrophages recovered from anti–LOX-1–treated mice (Figure 6E), consistent with our phenotype. However, we also observed a significant decrease in iNOS expression (Figure 6E), which is more aligned with an M1-like phenotype. We also did not detect any changes in MHCII (Figure 6E) or CD163 (data not shown) expression at the analyzed time points. Notably, at the same time point of 36 hours postinfection, when we observed reduced survival in anti–LOX-1–treated mice (Figure 2G), we also detected a trend toward fewer alveolar macrophages among the surviving mice, which may be due to inefficient replacement by monocyte-derived macrophages (Figure 6F). This possibility is further suggested by lower expression of CD11b (Figure 6E). Therefore, while alveolar macrophages underwent several phenotypic changes with anti–LOX-1 treatment, they were not strictly indicative of repolarization.

### Alveolar macrophages are transcriptionally reprogrammed following intrapulmonary LOX-1 neutralization.

**Figure 7:**
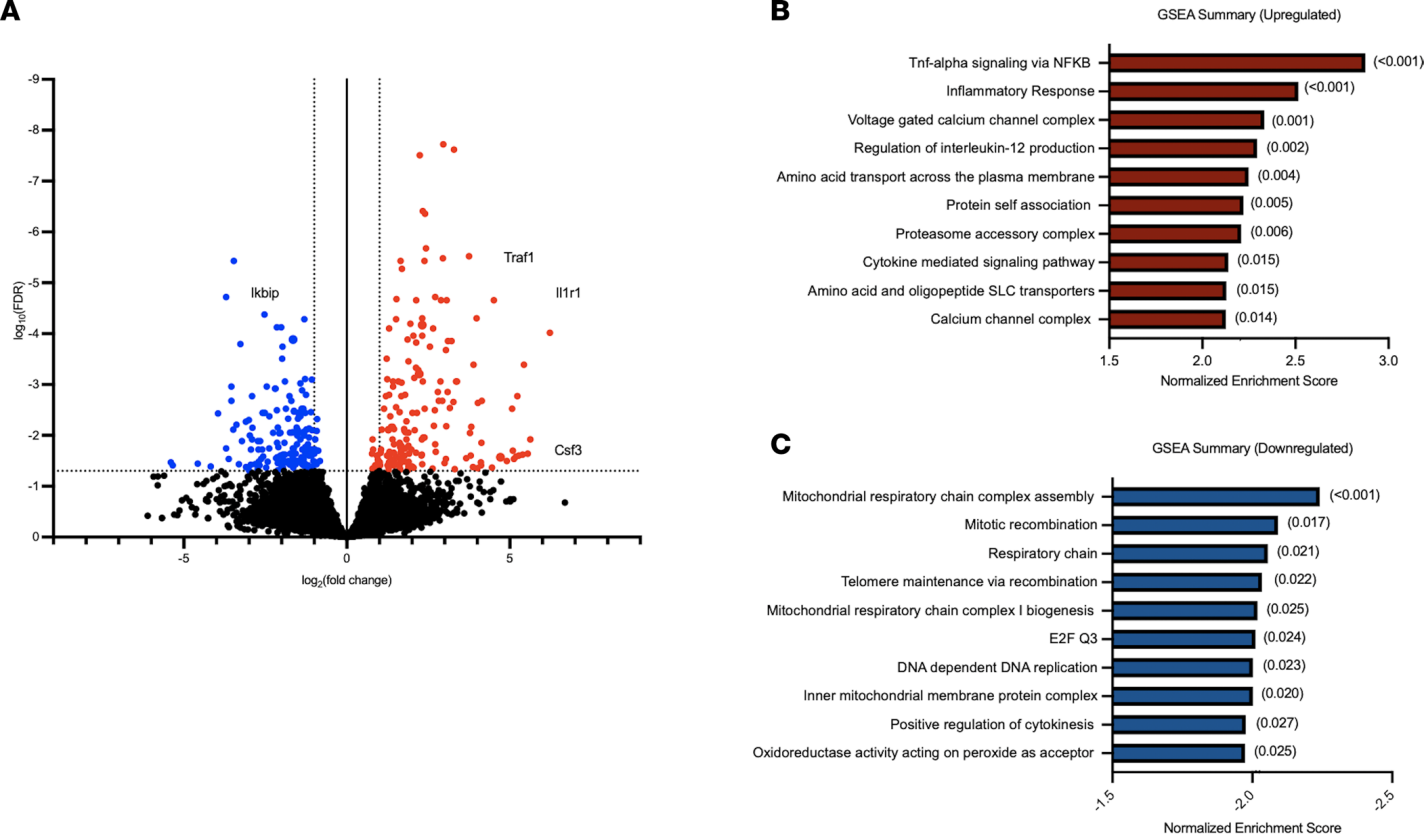
Alveolar macrophages are transcriptionally remodeled following LOX-1 blockade, resulting in elevated immune activity. Age-matched male and female C57BL/6 mice were intratracheally treated with 10 μg anti–LOX-1 IgG (*n* = 4) or control IgG (*n* = 4) and *E*. *coli* for 24 hours. FACS-sorted alveolar macrophages were analyzed by RNA sequencing. (**A**) A volcano plot was generated to illustrate all upregulated (FDR < 0.05, red) and downregulated (FDR < 0.05, blue) genes. (**B** and **C**) GSEA was performed on differentially expressed genes in macrophages isolated from anti–LOX-1– versus IgG-treated mice. The top 10 (**B**) upregulated and (**C**) downregulated pathways are displayed and ranked by normalized enrichment score with FDR values presented in parentheses.

Based on aforementioned results implicating important roles for LOX-1 on alveolar macrophages, we aimed to more comprehensively identify signaling pathways altered by LOX-1 blockade in this cell type, with the hypothesis that LOX-1 curbs immune activity as a means to limit inflammatory injury. To do so, we performed RNA sequencing on alveolar macrophages isolated from mice 24 hours after instillation with *E*. *coli* and either anti–LOX-1 Ab or IgG control. A total of 351 genes were differentially expressed (FDR *q* < 0.05), with 180 genes upregulated in anti–LOX-1–treated cells (red) and 171 genes that were downregulated (blue) (Figure 7A). Principal component analysis revealed significant separation of mice by Ab treatment in PC2, which explained approximately 15% of total variation (Supplemental Figure 9). Consistent with our hypothesis, many genes upregulated in anti–LOX-1–treated macrophages were canonical proinflammatory genes (*Il1r1*, *C1ql2*, *Csf3*, *Tlr2*, *Nlrp3*). In addition, *Cxcl2* tended (FDR *q* < 0.10) to be increased in anti–LOX-1–treated macrophages, a difference independently validated in FACS-sorted macrophages using RT-qPCR (Figure 6C). We also detected a multitude of intracellular signaling proteins that promote the NF-κB and MAPK pathways, such as *Irak3*, *Ticam2*, *Traf1*, *Traf5*, and *Traf6*. Interestingly, among genes significantly downregulated was IKBKB interacting protein (*Ikbip*), a protein that interferes with NF-κB signaling (Figure 7A), consistent with the observed increase for inflammatory gene programs. This shift toward dysregulated immunity was also supported by gene set enrichment analysis (GSEA), which included inflammatory response and TNF-α signaling via NF-κB among the top enriched pathways (Figure 7B) and mitochondrial respiratory chain complex assembly (Figure 7C) as the most downregulated within anti–LOX-1–treated macrophages. The latter may be attributable to the reliance of inflammatory processes on glycolytic rather than oxidative metabolism (36). Using Ingenuity Pathway Analysis (IPA, QIAGEN), we also identified potential upstream regulators based on the genes that were differentially expressed and their directionality. Again, in line with our hypothesis of exaggerated responses to lung infection, many of the top upstream regulators identified by IPA were associated with enhanced inflammation (TNF, IFNG, and IL1B) and infection with Gram-negative bacteria (TLR4) (Table 1). In sum, transcriptional profiling revealed enhanced immune reactivity in alveolar macrophages following LOX-1 blockade during pneumonia, implicating this cell type as a prominent source of cytokines and/or other factors underlying the heightened inflammatory injury elicited in the absence of LOX-1.

### Hematopoietic cells are the source of airspace LOX-1 during pneumonia and contribute to LOX-1–driven tissue protection.

**Figure 8:**
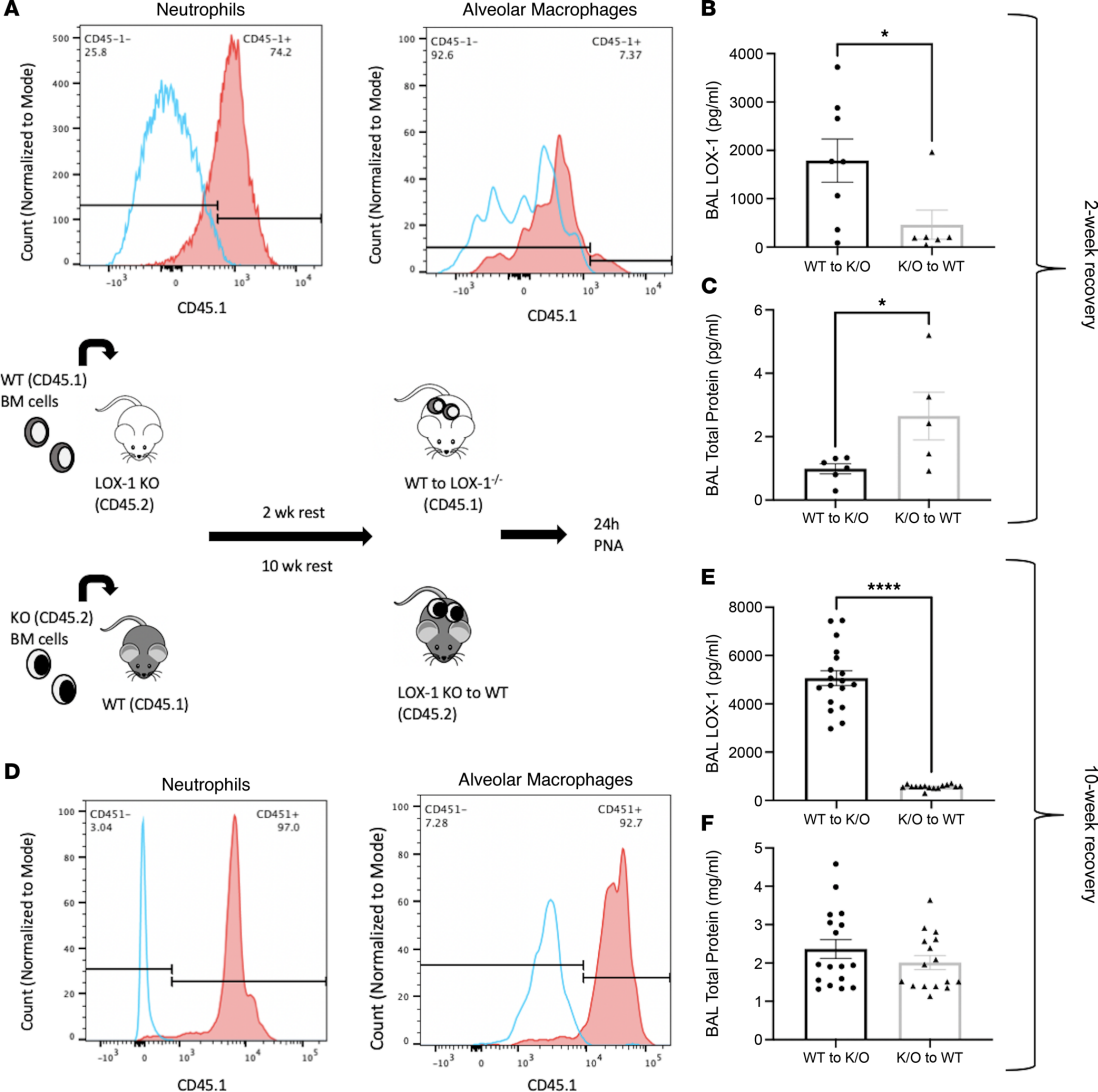
Hematopoietic cells are required for lung LOX-1 accumulation. (**A**) Bone marrow chimeras were generated from age-matched C57BL/6 (CD45.1) and LOX-1^–/–^ (CD45.2) mice and infected with *E*. *coli* for 24 hours at 2 weeks or 10 weeks after bone marrow engraftment (*n* = 5–17). Engraftment efficiency was measured in LOX-1^–/–^ mice at (**A**) 2 weeks and (**D**) 10 weeks posttransplant by measuring CD45.1 expression by flow cytometry on neutrophils and alveolar macrophages in BALF. (**B** and **E**) Soluble LOX-1 and (**C** and **F**) total protein (as a measure of injury) were determined in BALF following bone marrow transplant and intratracheal infection with *E*. *coli*. Data are represented as mean ± SEM with individual data points representative of mice from 2 independent experiments. ****P < 0.0001, *P < 0.05 for 2-tailed, unpaired *t* test (**B** and **C**) or 2-tailed Welch’s *t* test (**D**).

While LOX-1 inhibition resulted in more proinflammatory alveolar macrophages associated with elevated immunopathology, cell-specific contributions to the accumulation and protective effects of LOX-1 remain unclear. To delineate the roles of hematopoietic versus nonhematopoietic LOX-1 expression during pneumonia, we generated bone marrow chimeras using age-matched C57BL/6 WT (CD45.1) and LOX-1^–/–^ (CD45.2) mice as illustrated in Figure 8. Following irradiation and bone marrow transfer, the mice were allowed to recover for a 2- or 10-week period. After this time, mice were infected with *E*. *coli* for 24 hours, and percentage chimerism was assessed in neutrophils (CD45^+^CD11b^+^Ly6G^+^) and alveolar macrophages (CD45^+^CD11c^+^SiglecF^+^) by measuring CD45.1 expression as shown in Figure 8, A and D. These 2 recovery time points are sufficient to replace BALF neutrophils alone (2 weeks) or both neutrophils and macrophages (10 weeks). Using either strategy, BALF sLOX-1 accumulation was virtually ablated, with all but 1 sample at or near the limit of detection (Figure 8, B and E). This finding unequivocally identifies hematopoietic cells as the source of LOX-1 in the airspaces of the pneumonic lung. Importantly, proteinaceous edema was significantly increased in WT recipients receiving LOX-1–KO bone marrow (Figure 8C), supporting hematopoietic cells as not only the source of LOX-1 protein (Figure 8, B and E) but also the source of LOX-1–driven protection. However, the latter effect was limited to mice in which alveolar macrophages were not yet replaced, but this must be interpreted cautiously due to advanced age in the latter group, unknown manifestations of the irradiation protocol, and/or altered health or number of alveolar macrophages at the 2-week time point, possibly contributing to the changes in both LOX-1 and injury. Overall, we posit that alveolar macrophages are a predominant LOX-1 source in the resting lung (Figure 4), whereas the inducible pool of LOX-1 is driven by neutrophils and/or other hematopoietic sources, resulting in tissue protection.

### Recruited neutrophils contribute to the inducible LOX-1 pool in lungs during pneumonia.

**Figure 9:**
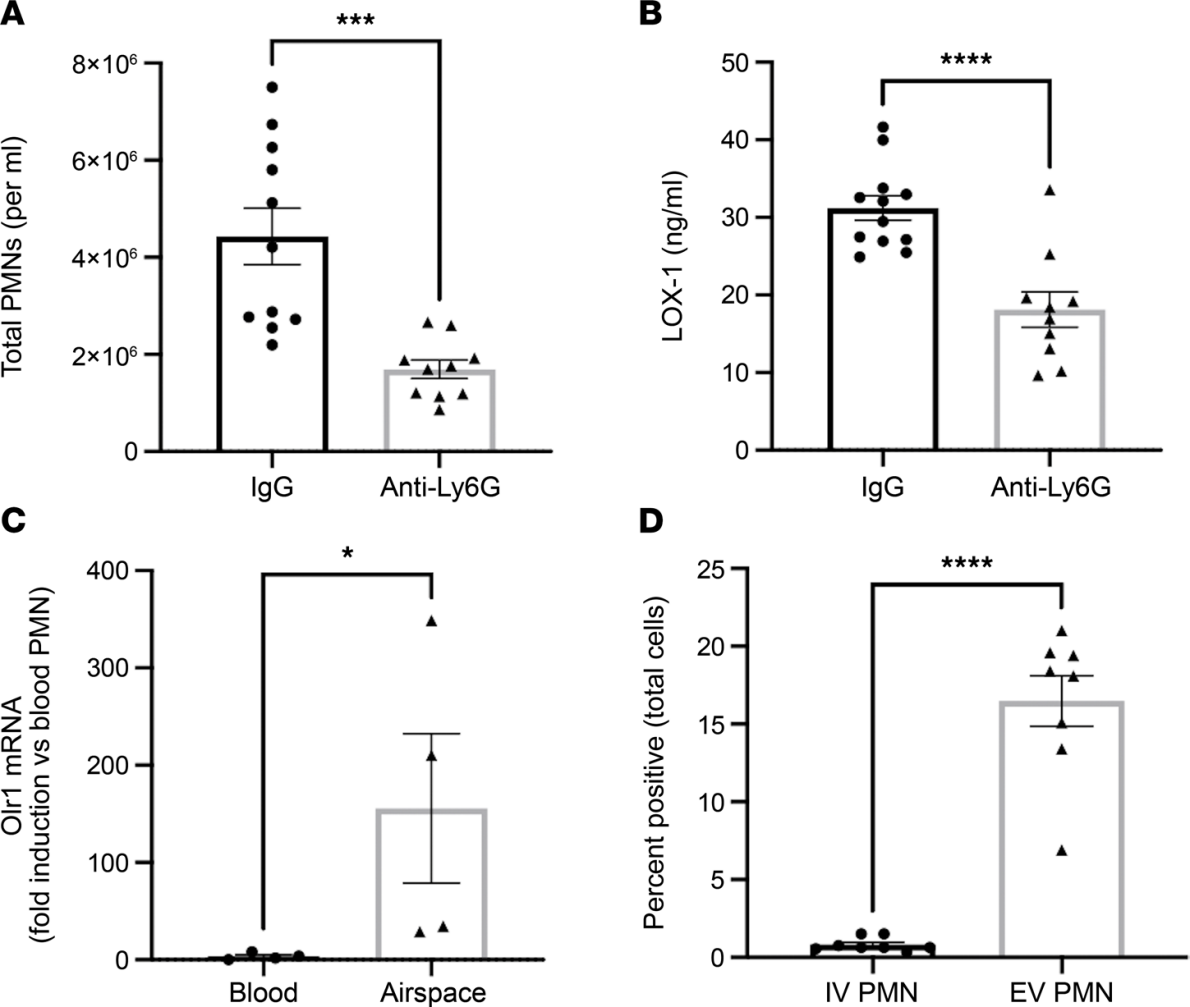
Airspace neutrophils are a significant source of lung LOX-1 during pneumonia. (**A** and **B**) Age-matched male and female C57BL/6 mice (*n* = 10–11) were intraperitoneally treated twice with 500 μg anti-Ly6G to deplete neutrophils and intratracheally infected with *E*. *coli* for 24 hours. (**A**) BALF neutrophil numbers and (**B**) lung homogenate LOX-1 concentrations were subsequently measured. (**C**) Gene expression of *Olr1* and (**D**) surface protein expression of LOX-1 was measured in sorted blood and airspace neutrophils (CD45^+^CD11b^+^Ly6G^+^) at 24 hours after intratracheal instillation with *E*. *coli*. Data are represented as mean ± SEM with individual data points representative of mice from 1–2 independent experiments. *P < 0.05, ***P < 0.001, ****P < 0.0001 for 2-tailed, unpaired *t* test (**A** and **B**) or ratio paired *t* test (**C** and **D**).

To identify whether neutrophils contribute to total lung LOX-1 accumulation during pneumonia, we depleted these cells using anti-Ly6G, which significantly but not completely reduced their recruitment into the lung (Figure 9A). This resulted in significantly lower lung LOX-1 concentrations (Figure 9B). Interestingly, we found that blood neutrophils expressed very little *Olr1* mRNA, but this outcome was dramatically induced in neutrophils that had extravasated to the lungs (Figure 9C). To confirm this result at the protein level, mice were infected for 24 hours with *E*. *coli*, and LOX-1 surface expression was compared on blood and airspace neutrophils. Although LOX-1 protein was only detected on a small fraction of circulating neutrophils, more than half of those recruited to the infected airspaces were LOX-1^+^, indicating that the pneumonic milieu is sufficient to elicit LOX-1 in this cell type (Figure 9D).

### LOX-1^+^ neutrophils are enriched for genes that regulate cholesterol metabolism and immunity.

**Figure 10:**
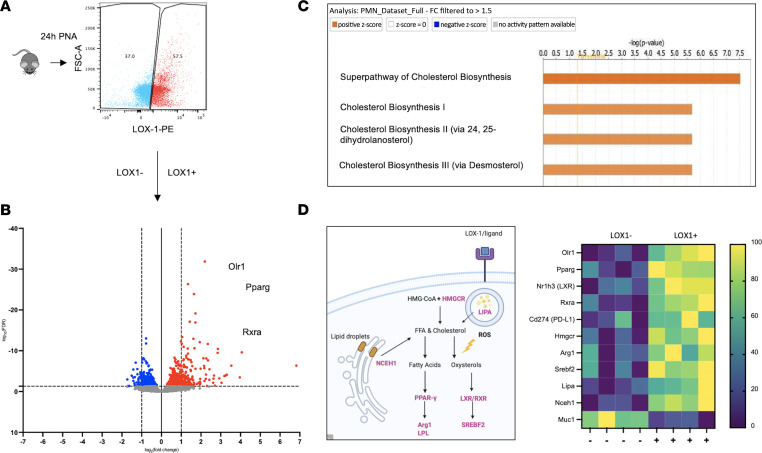
LOX-1^+^ neutrophils are transcriptionally distinct, with an enrichment of gene programs supporting cholesterol metabolism. (**A**) LOX-1^+^ and LOX-1^–^ neutrophils (CD45^+^CD11b^+^Ly6G^+^LOX-1^+^ or LOX-1^–^) were FACS-sorted 24 hours after intratracheal instillation of *E*. *coli* from age-matched male and female C57BL/6 mice (*n* = 4). (**B**) Cells were subsequently analyzed by RNA sequencing and were found (**C**) to be enriched with genes that regulate cholesterol handling by IPA. (**D**) Genes highlighted in purple and shown in the heatmap indicate genes that regulate cholesterol metabolism and inflammation and were significantly elevated in LOX-1^+^ neutrophils.

Measurement of LOX-1 among lung-recruited neutrophils revealed a wide range of surface expression, with nearly half lacking detectable levels of this receptor (Figure 10A). To determine whether LOX-1–expressing neutrophils are biologically distinct, we performed transcriptional profiling on LOX-1^+^ and LOX-1^–^ BALF neutrophils following 24 hours of pneumonia. Bulk RNA-Seq was performed to compare flow-sorted LOX-1^+^ neutrophils to LOX-1^–^ counterparts isolated from the same mice. Excitingly, 823 genes were differentially expressed in LOX-1^+^ neutrophils (FDR = *q* < 0.05), indicating a markedly distinct transcriptional profile aligned with the presence of this receptor (Figure 10B). Among the top genes were *Olr1* (as expected) and genes that promote antiinflammatory signaling (*Arg1*, *Cd274*, and *Pparg*). Moreover, IPA suggested a prominent role for cholesterol metabolism in LOX-1^+^ neutrophils, which is consistent with LOX-1 as a receptor for oxLDL (Figure 10C). LOX-1^+^ neutrophils also had higher expression of transcription factors RXR and PPARγ (Figure 10D), which are activated by oxysterols and polyunsaturated fatty acids, respectively (37, 38), and are important determinants of tissue repair and inflammatory regulation. Overall, these findings support that LOX-1 steers neutrophils toward a less inflammatory phenotype, consistent with the notion that LOX-1 expression in the pulmonary airspace limits immunopathology. Yet, the specific contributions of neutrophils, alveolar macrophages, and perhaps other unknown sources of LOX-1 remain unclear, constituting an important knowledge gap for future investigation.

## Discussion

Here we demonstrate substantial accumulation of LOX-1 and its ligands in the airspaces of both mice and humans with pneumonia, supporting a potentially novel precedent for LOX-1–mediated activity in response to respiratory infections. In contrast to other settings of inflammation, our findings also suggest that intrapulmonary LOX-1 serves a uniquely protective role, diminishing immunopathology without compromising antibacterial defense. While LOX-1 can be expressed by numerous cell types, our findings reveal hematopoietic cells as the prominent source in pneumonic airspaces. Prior to pneumonia, this receptor is remarkably prominent on alveolar macrophages and is even further expressed following infection. Alveolar macrophage responses are significantly exaggerated by LOX-1 blockade, implicating these cells as an important source of LOX-1–mediated immune regulation. Recruited neutrophils provide an additional source of lung LOX-1 following infection, possibly contributing to a less inflammatory gene program that serves as a countermeasure to limit pneumonia-induced immunopathology. To our knowledge, we are the first to identify LOX-1 accumulation in pneumonic lungs and the first to ascribe a tissue-protective function to this receptor.

LOX-1 was initially discovered as the primary receptor for oxLDL on endothelial cells, where it was shown to promote the development of vascular inflammation associated with the progression and severity of atherosclerosis (19). In the absence of inflammation, LOX-1 is lowly expressed; however, it is induced by numerous proinflammatory mediators, including but not limited to TNF-α, IL-1, oxLDL and other modified lipoproteins, endothelin 1, and angiotensin II (13). Moreover, its ligands extend beyond oxLDL to include several others, including activated platelets, apoptotic cells, heat shock proteins, CRP, live bacteria, bacterial products, and perhaps more (14). We found that LOX-1 and its ligands were elevated in pneumonic airspaces of both mice and humans. Regarding the former, it is notable that the soluble form of LOX-1 (as measured in BALF) may have functional roles distinct from its membrane-bound counterpart that were not addressed in this study. However, sLOX-1, which is cleaved by various enzymes and endopeptidases, such as ADAM17 and SPPL2a/b (15), has been used as a biomarker for vascular injury (16) and is also capable of interfering with oxLDL-induced injury (39), suggesting the exciting possibility that sLOX-1 tips the scale toward a more protective role in the lungs.

Prior literature convincingly reveals LOX-1 as a risk factor for incidence and severity of atherosclerosis, mediated by enhanced production of ROS, inflammatory cytokines, endothelial apoptosis, and recruitment of monocyte-derived macrophages into the intima, where they develop into foam cells (13). Moreover, LOX-1 decreases NO levels, thereby inhibiting vasodilation (40). Beyond atherosclerosis, LOX-1 promotes inflammation and injury in other conditions, such as systemic lupus erythematosus (41), diabetes mellitus (42), psoriasis (43), rheumatoid arthritis (20), and infection (44). However, little is known regarding the impact of LOX-1 on lung tissue, which has only been considered in the context of systemic inflammatory challenges. In those studies, LOX-1 interference diminished lung injury caused by systemic challenges with endotoxin and sepsis (via cecal ligation and puncture) (17, 22). As such, we anticipated that local suppression of LOX-1 in the lungs would dampen inflammatory injury in response to an intrapulmonary challenge with bacteria. Surprisingly, we found the opposite, with greater lung injury observed following neutralization of LOX-1 in the airspaces. A similar result was observed in WT mice reconstituted for 2 weeks with LOX-1–KO hematopoietic cells. While this was not recapitulated in global LOX-1–KO mice, our results suggest that if anything, global LOX-1 deficiency increases, rather than decreases, immune activity, at least based on neutrophil recruitment and bacterial clearance (Supplemental Figure 2). Importantly, while prior reports of LOX-1–induced lung injury (17, 22) differ from our own (indicating protection), we do not find them contradictory based on extremely different experimental circumstances. For instance, previous investigations determined consequences of *systemic LOX-1 targeting* (i.v. blocking Ab or global KO) in response to *systemic inflammatory challenges* (cecal ligation and puncture or i.p. LPS) (17, 22), contrasting our own, in which both the inflammatory stimulus and pharmacological LOX-1 blockade were initiated in the airspaces (i.t.). Taken together, these findings suggest opposing yet critical roles for intra- versus extrapulmonary LOX-1, wherein the pulmonary airspaces represent a niche for LOX-1–mediated protection.

To assess the potential sources of lung LOX-1 during pneumonia, we measured its surface expression and mRNA induction in both myeloid and nonmyeloid lung cells during pneumonia. In uninfected mice, alveolar macrophages were uniquely enriched for LOX-1 across all cell types analyzed, including other myeloid cells, such as dendritic cells, which can use LOX-1 as a mechanism for antigen presentation (45, 46). This led us to more specifically determine the effects of LOX-1 inhibition on alveolar macrophages, and our results indicated a dysregulated and heightened proinflammatory state based on transcriptional data from sorted cells. Here, we measured enhanced expression of pattern recognition receptors (*Tlr2*), adaptors that mediate NF-κB signaling (*Traf1*, *Traf5*, and *Traf6*) and *Nlrp3*, the sensor protein within the NLRP3 inflammasome, which, when activated, leads to the release of IL-1β and IL-18 (47). These changes are also reflected by elevated BALF cytokine levels and increased alveolar edema, suggesting that exaggerated macrophage activity disrupts tissue homeostasis in the absence of LOX-1. Downregulated genes were also consistent with our observed phenotype, whereby we measured a significant reduction in *Ikbip* (Figure 7A), a protein that inhibits NF-κB signaling through direct interaction with IKKα/β, resulting in lower phosphorylation of NF-κB (48). However, it is also possible that alveolar macrophage activity is secondary to changes in the surrounding environment since elevations in cytokines and injury could be observed as early as 12 hours after infection (Figure 2B and Supplemental Figure 4A). To address this possibility, we conducted additional experiments wherein WT and LOX-1–KO alveolar macrophages were exposed to an identical stimulus (WT pneumonic BALF) ex vivo. Our results again indicated an exaggerated response in the absence of LOX-1, supporting an intrinsic role for this receptor in immune regulation.

Based on these findings, we also determined whether LOX-1 blockade steers alveolar macrophages toward a more overtly polarized phenotype. Surprisingly, both proinflammatory (iNOS) and antiinflammatory markers (CD206 and Arginase 1) were reduced following LOX-1 blockade, supporting that LOX-1 influences the activity of this cell type, but not in a manner that directly aligns with M1- or M2-like polarization. Additionally, aside from CD206 (18 hours), differences in activation markers were relatively modest, occurring at a time point (36 hours) where significant mortality was already observed in anti–LOX-1–treated mice. Thus, results from this time point should be interpreted with caution.

Beyond polarization, alveolar macrophages have multiple mechanisms for resolving lung inflammation, some of which may be influenced by LOX-1 (49). For instance, efferocytosis, which involves the engulfment of dead and dying cells, is a prominent feature of alveolar macrophages and is also known to elicit an antiinflammatory, proresolving state (50). While perhaps less appreciated than its influence on vascular inflammation, LOX-1 has been identified as a facilitator of efferocytosis (51), perhaps contributing to the effects of LOX-1 blockade on both lung injury and macrophage activity, especially given the high LOX-1 expression on this cell type. Alternatively, engagement of LOX-1 on alveolar macrophages may initiate other tissue-protective and/or antiinflammatory signals through immune, metabolic, and/or other pathways that are yet to be determined.

While the aforementioned results implicate a major role for LOX-1 in alveolar macrophages, particularly under baseline homeostatic conditions wherein they are by far the most prominent for LOX-1 expression among lung cells, cellular sources of intrapulmonary LOX-1 remain an open question. To distinguish the specific contributions of hematopoietic cells, including alveolar macrophages, bone marrow chimeras were generated with WT and LOX-1^–/–^ mice. Excitingly, this study revealed that pneumonia-induced LOX-1 accumulation was solely driven by hematopoietic cells. Moreover, hematopoietic LOX-1 deficiency also exaggerated lung injury, again supporting an immune-regulatory role for LOX-1. While this particular phenotype (following 2 weeks of recovery) did not extend to our longer chimera protocol (10 weeks of recovery), experimental caveats such as advanced age, reduced numbers of macrophages 2-weeks posttransplant, and/or other manifestations of irradiation may be involved.

Regardless of the noted limitations, bone marrow chimera data convincingly identified neutrophils as a potential source of LOX-1 during pneumonia, especially given that these are the only other cells detected in the airspaces within the time frame of our study. Indeed, we found that neutrophil depletion markedly reduced lung LOX-1 accumulation during pneumonia, likely resulting from a robust increase in LOX-1 expression on a subset of emigrated neutrophils. Transcriptional profiling of LOX-1^+^ versus LOX-1^–^ neutrophils suggested that the former may have tamer immunological tone, possibly attributable to altered cholesterol metabolism and immunometabolic transcription factors known to limit immunopathology. For instance, metabolites produced during cholesterol metabolism, such as 25-hydroxycholesterol, have potent immunomodulatory properties, inhibiting IL-1β production and improving efferocytosis through activation of the liver X receptor (52). Moreover, LOX-1^+^ neutrophils exhibited higher expression of PPARγ, a transcription factor that prevents excessive injury in the lung during pneumonia (53). While the majority of current studies have focused on PPARγ in the context of macrophage biology, our data suggest that this transcription factor may also promote a resolving phenotype in neutrophils.

Overall, these studies reveal intrapulmonary LOX-1 as an important source of tissue resilience during pneumonia. While alveolar macrophages and neutrophils may be prominent sources of LOX-1–driven protection, additional studies are needed to precisely determine the cells and signals underlying the LOX-1 biology in the context of respiratory infection. Advances in this area could pave the way for novel clinical interventions in patients at risk for pneumonia.

## Methods

### Human samples.

BALF samples were collected by serial bronchoscopy from lungs of living patients enrolled in the Acute Lung Injury Specialized Center of Clinically Oriented Research randomized trial of granulocyte macrophage colony-stimulating factor administration from July 2004 through October 2007 at the Michigan Medicine Pulmonary Clinic at the University of Michigan. Patients were diagnosed with ARDS and compared with healthy control volunteers. ARDS inclusion criteria and patient demographic/clinical data have been described previously (54, 55). Lavage samples were shipped overnight, frozen on dry ice, and immediately stored at –80°C until further analysis.

### Mice.

C57BL6/J WT mice were purchased from Jackson Laboratory. LOX-1^–/–^ mice were provided by Shinshu University School of Medicine. Their generation has been described previously (19). Experiments were performed with both male and female mice 6–18 weeks of age and were age-matched within experiments. Mice were group-housed (≤5 mice/cage) and bred in a specific pathogen–free, temperature-controlled (20°C–23°C) environment, on a 12-hour light/12-hour dark cycle with food and water provided ad libitum. Littermates were randomized among experimental groups, and at least 3 mice were included in each experimental group. Exact mouse numbers per experiment are reported in figure legends. Experimental outcomes were blinded whenever possible. Experiments were performed at least twice, except where indicated in figure legends.

### Experimental pneumonia.

Mice were anesthetized with intraperitoneal (i.p.) injection of ketamine (50 mg/kg, Zoetis) and xylazine (5 mg/kg, Henry Schein Animal Health). Experimental pneumonias were induced by i.t. instillations of approximately 1 × 10^6^ to 3 × 10^6^ CFU *E. coli* (*E*. *coli* serotype 06:K2:H1; ATCC 19138; ATCC) or *S. pneumoniae* (*S*. *pneumoniae* serotype 3; ATCC 6303) diluted in sterile saline. I.t. instillations of bacteria or vehicle controls (saline) were directed into the left bronchus to achieve a lobar pneumonia as previously described (10). For experiments designed to test the effect of LOX-1 neutralization in the lungs, microbes were co-instilled with either 10 μg of anti–LOX-1 IgG or isotype control IgG (R&D Systems catalog AF1564 and AB-108-C). Effect of systemic LOX-1 blockade were tested by intravenous (i.v.) administration of 10 μg of anti–LOX-1 IgG or isotype control IgG immediately prior to i.t. instillation of bacteria. Mice were euthanized at various time points by isoflurane overdose (Henry Schein Animal Health).

### Alveolar macrophage depletion.

Alveolar macrophages were depleted as previously described by our group (11, 33), using 100 μL of clodronate (Liposoma; 5 mg of clodronate per mL of solution) or PBS-containing liposomes. Liposomes were delivered intranasally 72 hours prior to experimental pneumonia.

### Anti-Ly6G treatment.

Mice were treated i.p. with 500 μg anti-Ly6G or isotype control IgG2a (Bio X Cell clone 1A8 and 2A3) at –24 hours and 0 hours prior to i.t. instillation with *E*. *coli* for 24 hours. Blood, frozen lungs, and BALF were subsequently collected.

### Bone marrow chimera generation.

Bone marrow chimeras were generated from WT (CD45.1) or LOX-1^–/–^ mice, distinguished from one another based on the CD45.1 versus CD45.2 allele, similar to previous studies in our laboratory (11). Lethal irradiation (X-RAD 320; Precision X-ray) was performed 4 hours prior to bone marrow transplant using a dose of 13 Gy, split into 2 doses of 6.5 Gy administered 4 hours apart. Bone marrow cells were isolated from the femur and tibia of mice, collected in sterile PBS, and filtered through a 70 μm filter (Thermo Fisher Scientific). Approximately 5 × 10^6^ cells, delivered in 100 μL sterile PBS, were transplanted to each mouse with a retroorbital injection. Chimeric mice were allowed to reconstitute their hematopoietic cells for either 2 or 10 weeks posttransplant. Following recovery, all mice were infected i.t. with *E*. *coli* for 24 hours. BALF was collected for total protein and sLOX-1 analysis. BALF and blood cells were collected to confirm chimerism.

### Lung harvest and bronchoalveolar lavage.

BALF was collected using 10 washes (1 mL each) with PBS as previously described (11, 56). Lavaged lungs were snap-frozen in liquid nitrogen and stored at –80°C. Total cell counts were obtained from a LUNA-FL dual fluorescence cell counter (Logos Biosystems), and differential cell counts were performed manually on cytospin slides stained with Diff-Quick (VWR). Total protein in BALF was determined by bicinchoninic acid (BCA) assay (MilliporeSigma).

### Alveolar macrophage culture.

Primary alveolar macrophages were collected from WT and LOX-1^–/–^ mice by lavaging lungs with RPMI supplemented with 10% fetal bovine serum (FBS; MilliporeSigma) and 1% penicillin/streptomycin (Gibco). Alveolar macrophages were spun down at 300*g* for 5 minutes at 4°C and enumerated using the LUNA-FL cell counter. Approximately 1 × 10^5^ cells/well were immediately stimulated with cell-free lavage fluid collected from uninfected mice or mice i.t. infected with *E*. *coli* for 24 hours, diluted 1:2 with complete RPMI. Cells were treated for 4 hours at 37°C, 5% CO_2_ in a humidified incubator. After 4 hours, medium was removed, and cells were immediately lysed with RLT lysis buffer (QIAGEN). Lysates were frozen at –80°C for downstream analysis.

### Western blot.

Snap-frozen lungs were homogenized in protein extraction buffer containing complete protease inhibitor (Roche) using a Bullet Blender (Next Advance) as previously described (10). Protein concentrations were determined by BCA assay, and equal amounts of protein were loaded onto a NuPAGE 4%–12% Bis-Tris gel (Thermo Fisher Scientific) and transferred to an Immobilon-P PVDF (MilliporeSigma) using the X-Cell Blot II system. Proteins were detected by probing with anti–LOX-1 (R&D Systems catalog AF1564), anti-GAPDH (Cell Signaling Technology clone 14C10) or anti–Pan-Actin (Cell Signaling Technology clone D18C11) followed by anti-rabbit–HRP (Cell Signaling Technology catalog 7074), anti-goat–HRP (MilliporeSigma; catalog AP106P), anti-goat IRDye 680RD (LI-COR Biotechnologies catalog 926-32214)/800CW (LI-COR Biotechnologies catalog 926-68074), or anti-rabbit IRDye 680RD (LI-COR Biotechnologies catalog 926-68071)/800CW (LI-COR Biotechnologies catalog 926-32211) and developed with ECLPlus (GE Healthcare, now Cytiva) before film exposure (GE Healthcare) or visualized on the Odyssey CLx Imaging System (LI-COR Biotechnologies).

### ELISA and cytokine determination.

LOX-1, CRP, IL-6, CXCL2, and oxLDL concentrations in mouse and human BALF were determined using ELISA, per manufacturer’s instructions (R&D Systems; sLOX-1, CRP, IL-6, and CXCL2) (CUSABIO; murine oxLDL) (Mercodia; human oxLDL). LDH was measured using the CytoTox 96 Non-Radioactive Cytotoxicity Assay (Promega) per manufacturer’s instructions. Additional cytokines were measured using a mouse magnetic Luminex assay (R&D Systems) and read on a LiquiChip 200 workstation (Qiagen).

### Bacteriology.

After euthanasia, infected left lobes were immediately homogenized using a Bullet Blender (Next Advance) in sterile distilled water. Homogenates were serially diluted and grown on 5% sheep blood agar plates (BD Biosciences). After overnight incubation at 37°C, 5% CO_2_, colony numbers were counted and CFU calculated as total CFU per lung.

### Lung histology and immunofluorescence.

Lungs were prepared for histology and immunofluorescence as previously described (57, 58). Frozen sections (8 μM) were fixed, permeabilized with 0.2% Triton-X, and blocked with 10% donkey serum and 3% bovine serum albumin (blocking buffer). Sections were then incubated overnight at 4°C in a humidified chamber with the following primary Abs: α-F4/80 (1:100, catalog 6640) and α–LOX-1 (1:100, catalog 60178) (Abcam). Sections were then washed and incubated with the following secondary Abs: Alexa Fluor 488–conjugated AffiniPure donkey anti-rat IgG (1:1,000, catalog 712-545-150) and Alexa Fluor 594–conjugated AffiniPure donkey anti-rabbit IgG (1:1,000, catalog 711-585-152) (Jackson Immunoresearch) at room temperature for 1 hour in a dark, humidified chamber. Secondary-only controls for LOX-1 (no α–LOX-1) and F4/80 (no α-F4/80) were also generated. Slides were then washed and counterstained with DAPI (Life Technologies) and mounted with FluorSave (MilliporeSigma). Visualization of histology and immunofluorescence was performed on a Leica DM4 LED light microscope equipped with a Leica DFC 7000T camera. Images were taken on dry, coverslipped slides using a 40× objective (total magnification = 400×) at room temperature with a numerical aperture of 0.8 using the Leica Application Suite X software. Images were processed using ImageJ 2.0.0-rc-69 (NIH).

### Lung cell suspensions.

Lungs were digested in elastase, dispase, or collagenase. For all digestion procedures, lungs were perfused through the right ventricle with 10 mL cold HBSS (Thermo Fisher Scientific). For elastase and dispase digests, the heart-lung block was then removed and lavaged with 10 mL Dulbecco’s PBS–EDTA. Lungs were digested with a mixture of 4.5 U elastase or 10 U dispase (Worthington Biochemicals), 10% dextran (MilliporeSigma), 150 μg/mL DNase (MilliporeSigma), and 0.5 mL 1% low melting temperature agarose (MilliporeSigma), all dissolved in RPMI as previously described (11). For myeloid cell isolation, the heart-lung block was removed, and both lobes were gently minced in digestion solution (1× PBS, 1 mg/mL type II collagenase from Worthington Biochemicals, 150 μg/mL DNase from MilliporeSigma, 2.5 mM CaCl_2_) as previously described (59).

To distinguish intra- versus extravascular cells within the same mouse, α-CD45.2-PercpCy5.5 (10 μg/mL; clone 104, BioLegend) was intravenously delivered via retroorbital injection 3 minutes prior to euthanasia to stain circulating cells. Nonperfused, nonlavaged left (sample) and right (FMO controls) lobes were then removed and processed using the same myeloid digestion protocol as above. A mouse without α-CD45.2 i.v. treatment was used as the FMO control for the i.v. stain. Following all digestion protocols, erythrocytes were lysed with a red blood cell lysis buffer (MilliporeSigma). Cells were then counted using the LUNA-FL dual fluorescence cell counter (Logos Biosystems).

### Flow cytometry and cell sorting.

After enumeration, cells were resuspended in Fc receptor blockade (FcBlock, eBioscience) and stained with monoclonal Abs for 30 minutes (4°C, in dark). The following fluorochrome-conjugated monoclonal Abs were used: CD45-PECy7 (clone 30-F11, BD Biosciences), CD45-BV510 (clone 30-F11, BD Biosciences), CD45-FITC (clone 30-F11, eBioscience), CD45.1-PECy7 (clone A20, BioLegend), CD45.2-BUV737 (clone 104, BD Biosciences), CD31-FITC (clone MEC 13.3, BD Biosciences), EpCAM-APC (CD326, clone G8.8, eBioscience), F4/80-BV421 (clone BM8, BioLegend), Ly6G-APCCy7 (clone 1A8, eBioscience), Ly6G-APC (clone 1A8, BD Biosciences), Ly6G-BUV737 (clone 1A8, BD Biosciences), CD11b-BUV395 (clone M1/70, BD Biosciences), CD11c-PECy7 (clone HL3, BD Biosciences), CD64-FITC (clone X54-5/7.1, BioLegend), SiglecF-APCCy7 (clone E50-2440, BD Biosciences), MHCII-PercpCy5.5 (clone M5/114.15.2, BD Bioscience), Ly6C-eFluor 450 (clone HK1.4, eBioscience), and LOX1-PE (clone 214012, R&D Systems). Viability staining was performed using 7-AAD Viability Staining Solution (BioLegend). ROS were measured using the CellROX deep red reagent (Thermo Fisher Scientific) per manufacturer’s instructions. Flow cytometry data were acquired using the BD LSR II flow cytometer and the BD FACSDiva software (both BD Biosciences). Cell sorting experiments were done using the BD FACSAria II SORP cell sorter (BD Biosciences). Fluorescence compensation was performed with single-stained UltraComp eBeads (eBioscience), unstained cells, and 50% heat-killed cells singly stained with 7-AAD. FMO controls were used in all experiments to gate positive staining.

### Intracellular flow cytometry.

Cells were resuspended in 1× PBS containing Zombie Violet (BioLegend). Viability staining was performed for 30 minutes (room temperature, in dark). After washing, cells were resuspended in Fc receptor blockade (eBioscience) and stained with monoclonal Abs for 30 minutes (4°C, in dark). Cells were washed and fixed in 4% paraformaldehyde for 20 minutes on ice in the dark. Cells were washed twice and held at 4°C overnight. Cells were then permeabilized with 1× BD Perm/Wash (BD Biosciences) for 20 minutes (4°C, in dark) and stained for intracellular antigens in 1× Perm/Wash buffer for 30 minutes (4°C, in dark). In addition to those above, the following fluorochrome-conjugated monoclonal Abs were used: Arginase-1-APC (clone A1exF5, eBioscience), CD206-PE/Dazzle (clone C068C2, Biolegend), iNOS-PE (clone CXNFT, eBioscience), and CD163-Super Bright 600 (clone TNKUPJ, eBioscience). Cells were washed twice with 1× Perm/Wash buffer and resuspended in FACS buffer prior to analysis. Flow cytometry data were acquired as above. Fluorescence compensation was performed with single-stained UltraComp eBeads, unstained cells, and 50% heat-killed cells singly stained with Zombie Violet. FMO controls were used in all experiments to gate positive staining.

### RNA extraction and RT-qPCR.

FACS-sorted cells were collected in RPMI containing 10% FBS, immediately centrifuged (300*g*, 5 minutes, 4°C), and resuspended in RNAprotect (QIAGEN) or RLT lysis buffer supplemented with 2-mercaptoethanol. Cell lysate was homogenized using a Qiashredder (QIAGEN) and extracted using the RNeasy Micro Kit (QIAGEN) per manufacturer’s instructions. RT-qPCR was done on a QuantStudio 3 Real-Time PCR System (Thermo Fisher Scientific) using the TaqMan RNA-to CT one-step kit (Thermo Fisher Scientific). The following commercially-available FAM-labeled primer and probe sets were used: *Olr1* (Mm00454586_m1), *Il6* (Mm00446190_m1), and *Cxcl2* (Mm00436450_m1) (Applied Biosystems). These were used in conjunction with VIC-labeled eukaryotic 18S rRNA endogenous control (Life Technologies). Expression values are presented as fold induction and have been corrected for 18S rRNA expression.

### RNA sequencing.

Alveolar macrophages from anti–LOX-1– and IgG-treated mice along with LOX-1^+^ and LOX-1^–^ neutrophils (separate experiment) were isolated by FACS, and RNA was extracted using the RNA Micro Kit (QIAGEN). RNA quality was assessed on a Bioanalyzer (Agilent Technologies), and an RNA integrity number score > 8.0 was used as inclusion criteria. Four mice per group over 2 independent experiments were used for analysis. Samples were sequenced using the Illumina NextSeq 500 system, and FastQC files were aligned to the mm10 genome using STAR (version 2.6.0c). Ensembl-Gene-level counts were generated for nonmitochondrial genes using featureCounts (Subread package, version 1.6.2) and Ensembl annotation build 99 (uniquely aligned proper pairs, same strand). In addition to count reads aligning to proper pairs at least once to either strand of the mitochondrial chromosome or to sense or antisense strands of Ensembl loci of gene biotype “rRNA” or of nonmitochondrial RepeatMasker loci of the class “rRNA” (as defined in RepeatMasker track retrieve from the UCSC Table Browser), we used SAMtools (version 1.9). The quality of FASTQ files was determined using FastQC (version 0.11.7), and alignment quality was determined by RSeQC (version 3.0.0). Original sequencing files have been deposited to the NCBI Gene Expression Omnibus under Series ID GSE208233.

### Statistics.

Statistical analyses were performed in Prism V.8 (GraphPad Software). Data are presented as mean ± SEM. Sample size in each experiment is detailed in the figure legend and displayed within each graph, where *n* = number of mice or human patients. Data points are indicative of biological replicates, with the exception of the survival curve, which is displayed as summary data. Normality of data was determined using a Shapiro-Wilk test. Data that were not normally distributed were either analyzed using nonparametric tests or were log-transformed prior to analysis. Statistical tests used are described in the figure legends. Differences were considered significant if *P* < 0.05, and the following represents the level of significance: ****P < 0.0001, ***P < 0.001, **P < 0.01, and *P < 0.05.

Differential expression analysis for RNA-sequencing data was performed using a Wald test in the DESeq2 R package (version 1.22.1). A Benjamini-Hochberg FDR correction was applied to determine FDR-corrected *P* values (*q* value). Additionally, *q* values were also determined after removing genes that did not pass an “independent filtering” step in the DESeq2 package. Principal component analysis was performed using the *prcomp* R function with variance stabilized transformed expression values that were *z*-normalized across all samples within each gene. Human homologs for each gene were identified using HomoloGene (version 68). All analyses were performed using the R environment for statistical computing (version 3.5.1). Enriched pathways and upstream regulators were determined using IPA (Qiagen) of all genes that were differentially expressed with *q* < 0.05 and fold change > 1.5.

### Study approval.

Animal procedures were approved by the Institutional Care and Use Committees at Boston University and UMass Chan Medical School (protocol no. PROTO201800710 and PROTO202100149, respectively). Studies involving human participants were approved by the University of Michigan Institutional Review Board. Written informed consent by each patient or legal proxy for medical decision was a requirement for study inclusion.

## Author contributions

LJQ devised this study and provided expertise with data analysis and interpretation and manuscript preparation. FTK designed and performed experiments, analyzed data, and wrote the manuscript. ATS assisted with experimental design, implementation, and analysis. EMS and LAB performed select experiments and provided mouse genotyping. CVO, ELD, and WMA performed select experiments. EIA aided with myeloid cell flow cytometry. MB provided cell sorting. TJS supplied human samples. JLM provided expert consultation and LOX-1 small molecule inhibitor. TS provided the LOX-1–KO mouse. MRJ, JPM, and KET advised on experimental design and interpretation on a regular basis. Additionally, JPM and KET contributed financially. FTK, ATS, EMS, LAB, CVO, EIA, ELD, EN, WMA, MB, TJS, JLM, TS, MRJ, JPM, KET, and LJQ assisted with data analysis and presentation and manuscript revision.

## Supplementary Material

Supplemental data

## Figures and Tables

**Table 1:**
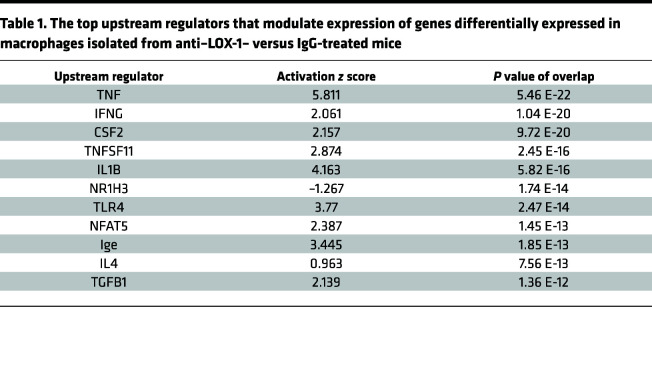
The top upstream regulators that modulate expression of genes differentially expressed in macrophages isolated from anti–LOX-1– versus IgG-treated mice

## Version 1 10/20/2022

In-Press Preview

## Version 2 12/08/2022

Electronic publication

## Version 3 12/13/2022

Revised author notes section
