# Supplementary material for: Lectin-like oxidized low-density lipoprotein receptor 1 attenuates pneumonia-induced lung injury
Source: JCI Insight. 2022 Dec 8;7(23):e149955. doi: 10.1172/jci.insight.149955 (PMC9746901; doi:10.1172/jci.insight.149955)
Supplement: Supplemental data [file jciinsight-7-149955-s273.pdf]

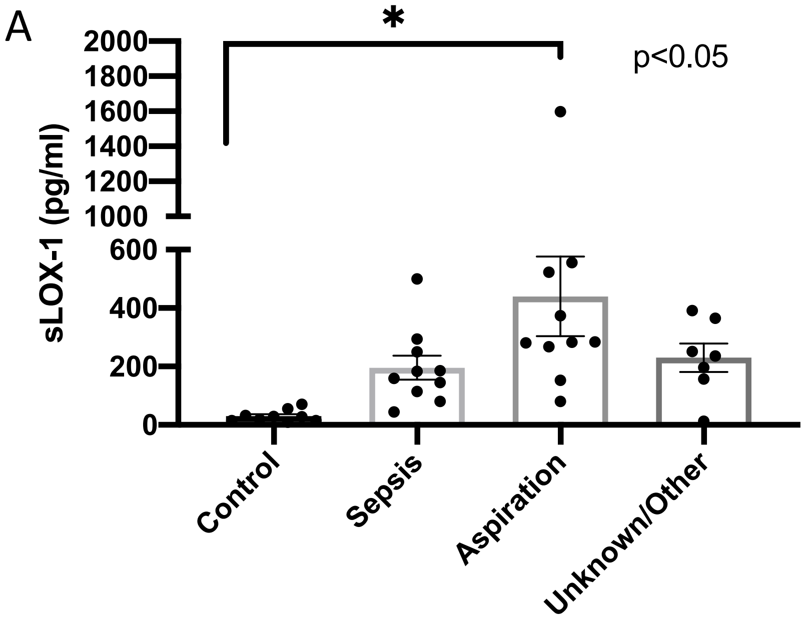

Figure S1: Soluble LOX-1 is induced in bronchoalveolar lavage under conditions of non-pneumonia ARDS in humans. BALF was obtained from human patients with ARDS as a result of aspiration (n=10), a confirmed diagnosis of sepsis (n=10), or of either unknown (n=3) or less common causes, such as post-op (n=1), transfusion (n=2), pancreatitis (n=1) and non-ARDS healthy controls (n=9) and LOX-1 protein was measured. Data are presented as mean ± SEM with individual data-points representative of different patients. *$p<0.05$ for one-way ANOVA with Dunnett's post-hoc test.

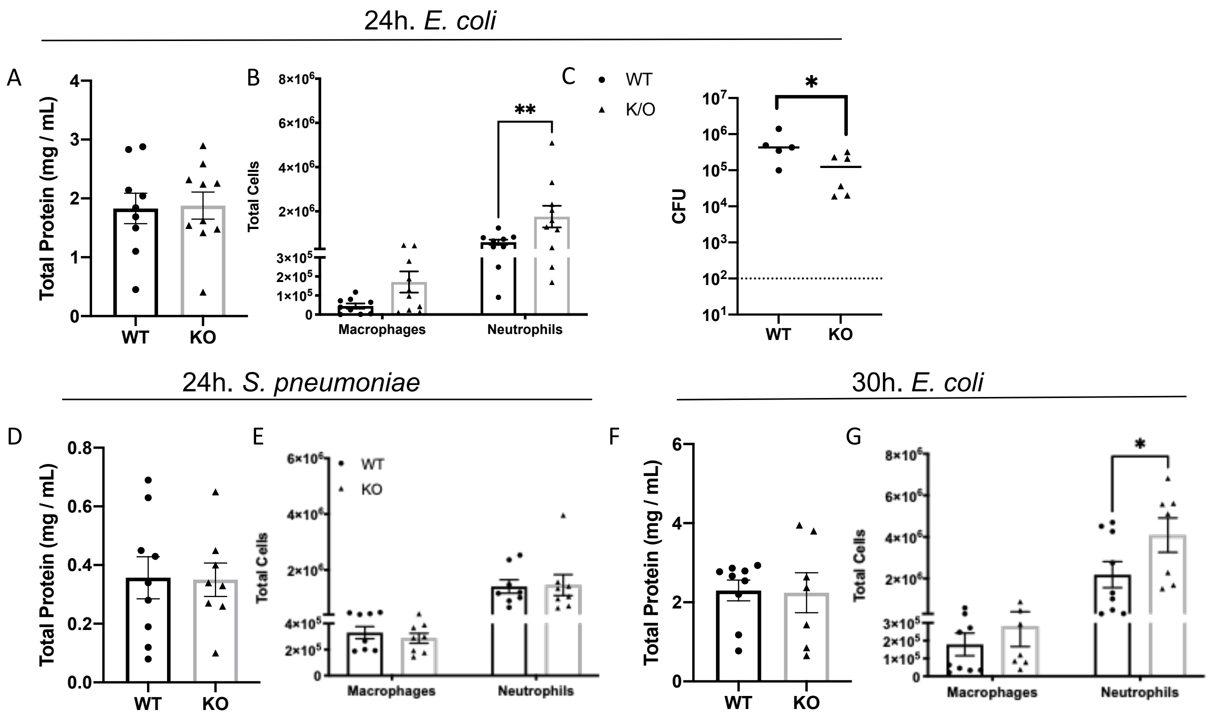

Figure S2: LOX-1 knockout mice exhibit enhanced leukocyte influx with a concomitant decrease in bacterial growth. Age-matched LOX-1 knockout and WT C57BL/6 mice (n=5-9 per group) were intratracheally infected with *E. coli* (**A-C**) or *S. pneumoniae* **(D-E)** for 24h and *E. coli* for 30h (**F-G**). Total protein (**A, D & F**) and differential leukocyte numbers (**B, E & G**) were measured in BALF. (**C**) Lung CFU was measured in *E. coli* infected mice at 24h post infection. Data are represented as mean ± SEM with individual data-points representative of mice from 2-3 independent experiments. CFU data was log-transformed and statistics were performed on transformed values (dotted line = limit of detection). ** $p<0.01$, *$p<0.05$ for two-way ANOVA and Sidak's post-hoc test (B & G) or unpaired, two-tailed t-test (C).

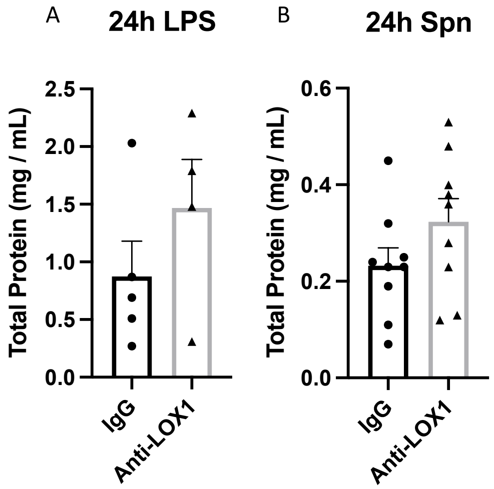

Figure S3: Lung injury is unaffected by LOX-1 blockade and intratracheal *S. pneumoniae* or LPS. Age-matched C57BL/6 mice were treated intratracheally with 10 µg anti-LOX-1 or control IgG and either (**A**) *S. pneumoniae* or (**B**) LPS. Total protein was subsequently measured in BAL at 24h post intratracheal instillation.

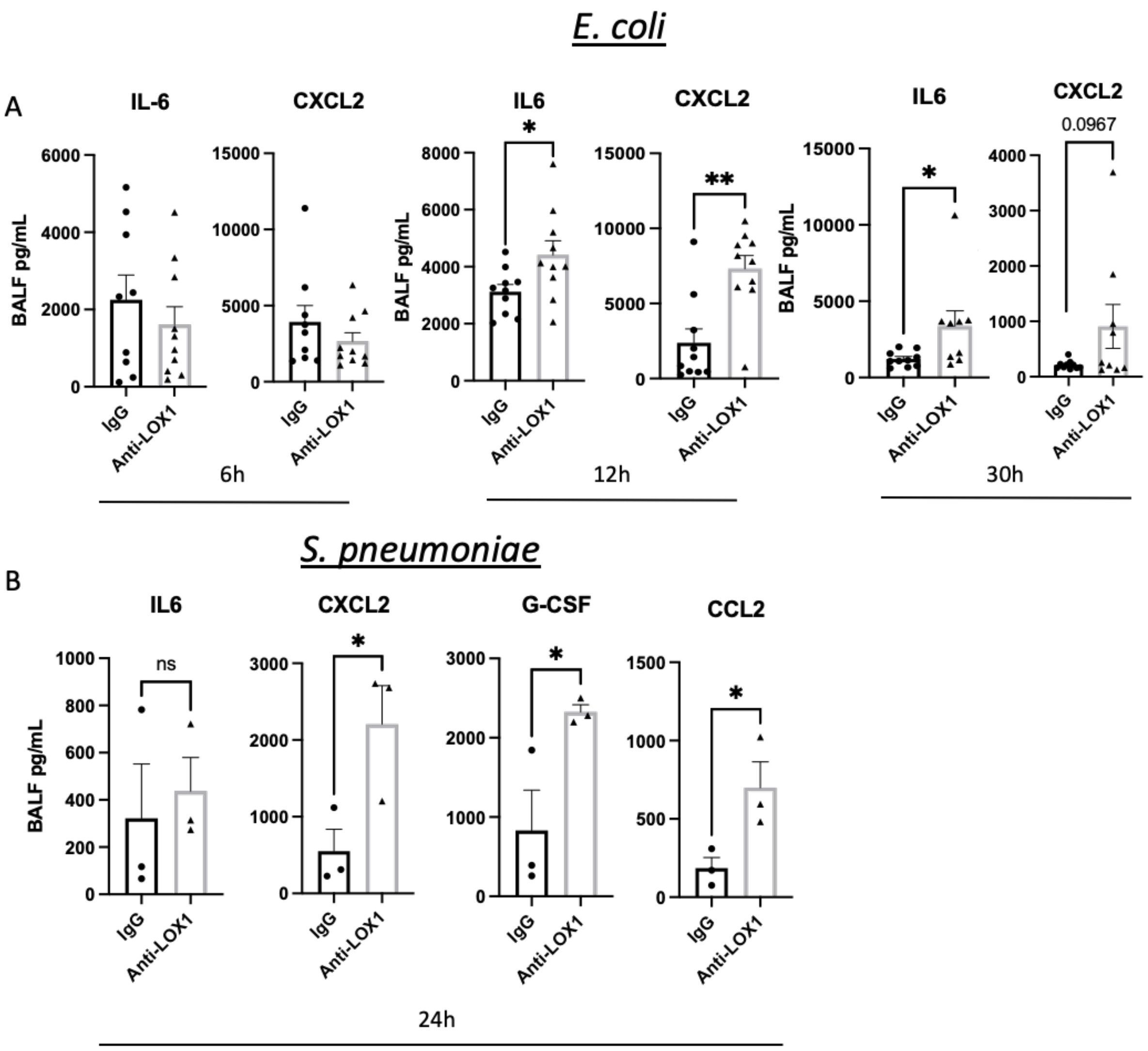

Figure S4: Increased inflammation is observed at varying time points post-infection with LOX-1 blockade. Age-matched C57BL/6 mice were treated intratracheally with 10 µg anti-LOX-1 or control IgG and either (**A**) *E. coli* or (**B**) *S. pneumoniae* and cytokine concentrations were determined in BAL at 6, 12 and 30h post-infection in *E. coli* treated mice and at 24h post-infection in *S. pneumoniae* treated mice. Data are represented as mean ± SEM with individual

data-points representative of mice from 2-3 independent experiments. **p<0.01, *p<0.05 for two-tailed, unpaired t-test.

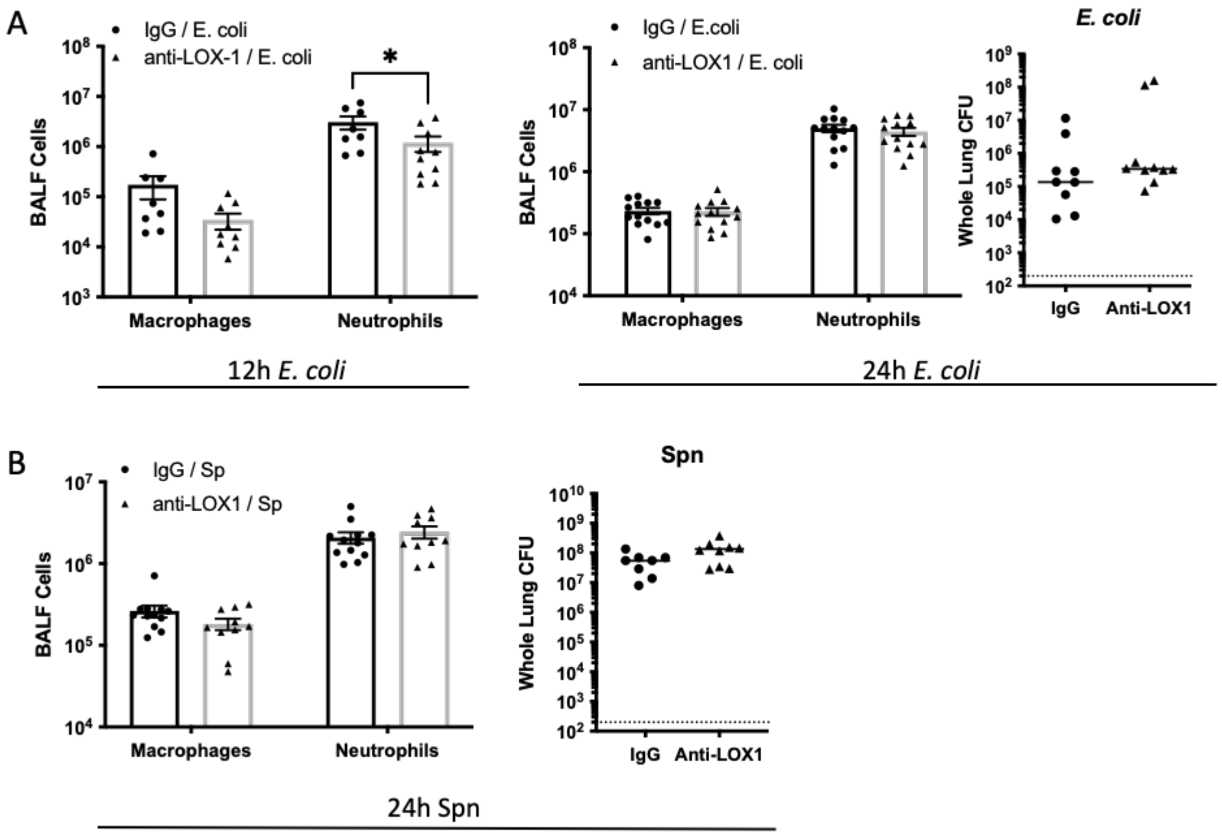

Figure S5: No change in BALF cellularity or bacterial CFU is detected in mice post-infection with LOX-1 blockade.

BAL macrophages and neutrophils were measured at 12-24h post-infection with (A) *E. coli* and 24h post-infection with (B) *S. pneumoniae.* Lung CFU was measured at 24h post-infection with (A) *E. coli* and (B) *S. pneumoniae.* Data are represented as mean ± SEM with individual data-points representative of mice from 2-3 independent experiments. *p<0.05 for two-tailed, unpaired t-test.

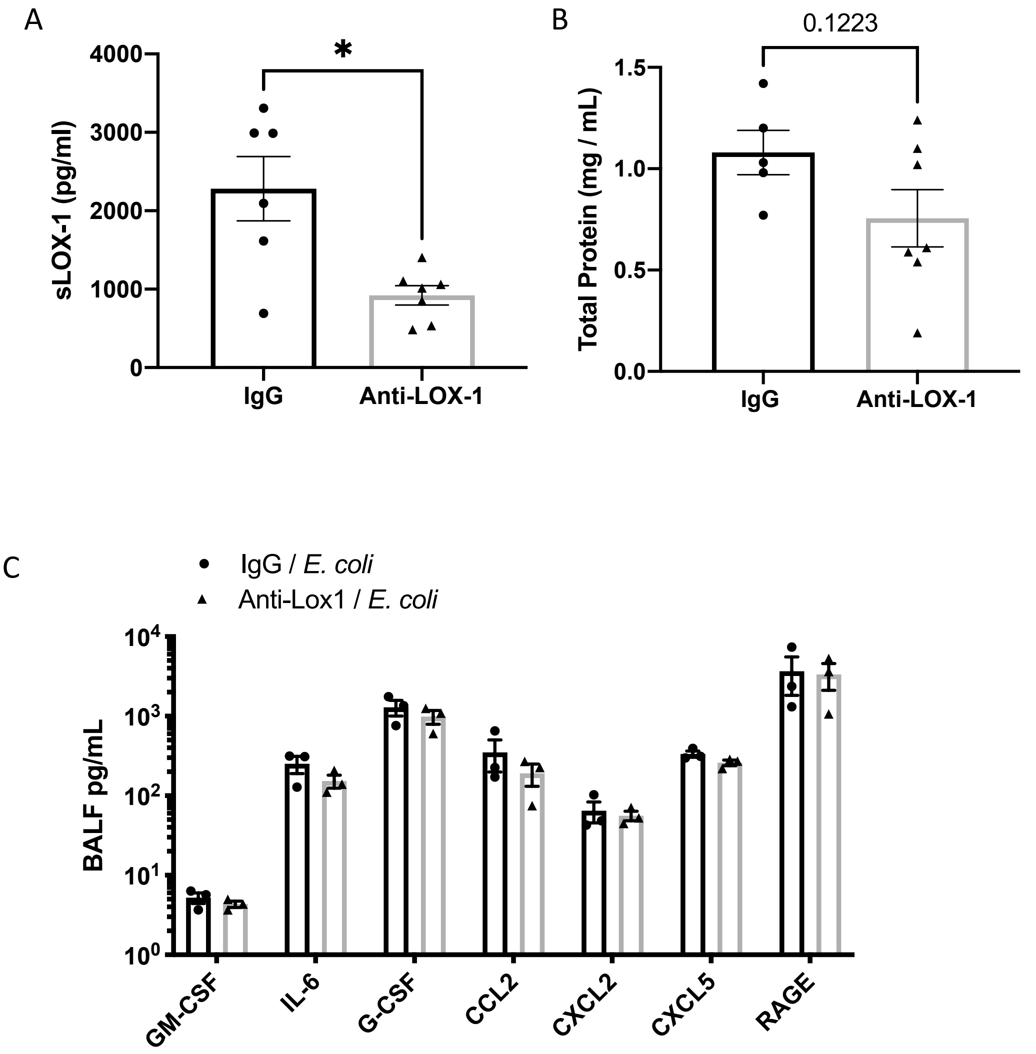

Figure S6: Intravenous LOX-1 neutralization does not protect from lung injury. (A-D) Age-matched C57BL/6 mice (n=3-7 per group) were treated intravenously with 10 μg anti-LOX-1 or control IgG immediately prior to intratracheal infection with *E. coli.* BALF (**A**) soluble LOX-1, (**B)** total protein and (**C**) cytokine concentrations were measured. Data are represented as mean ± SEM with individual data-points representative of mice from 2-3 independent experiments. *p<0.05 for two-tailed, Welch's t-test.

Intra vs. Extravascular Cell Gating

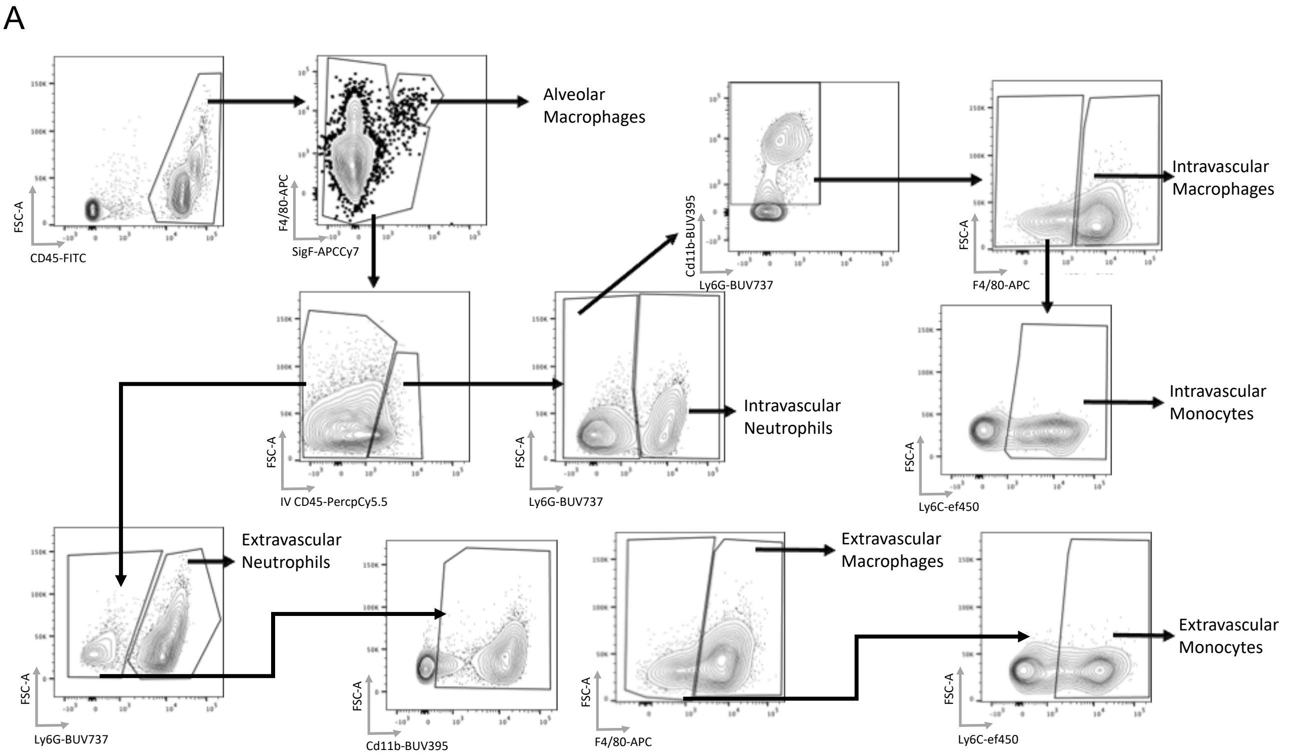

Myeloid Cell Gating

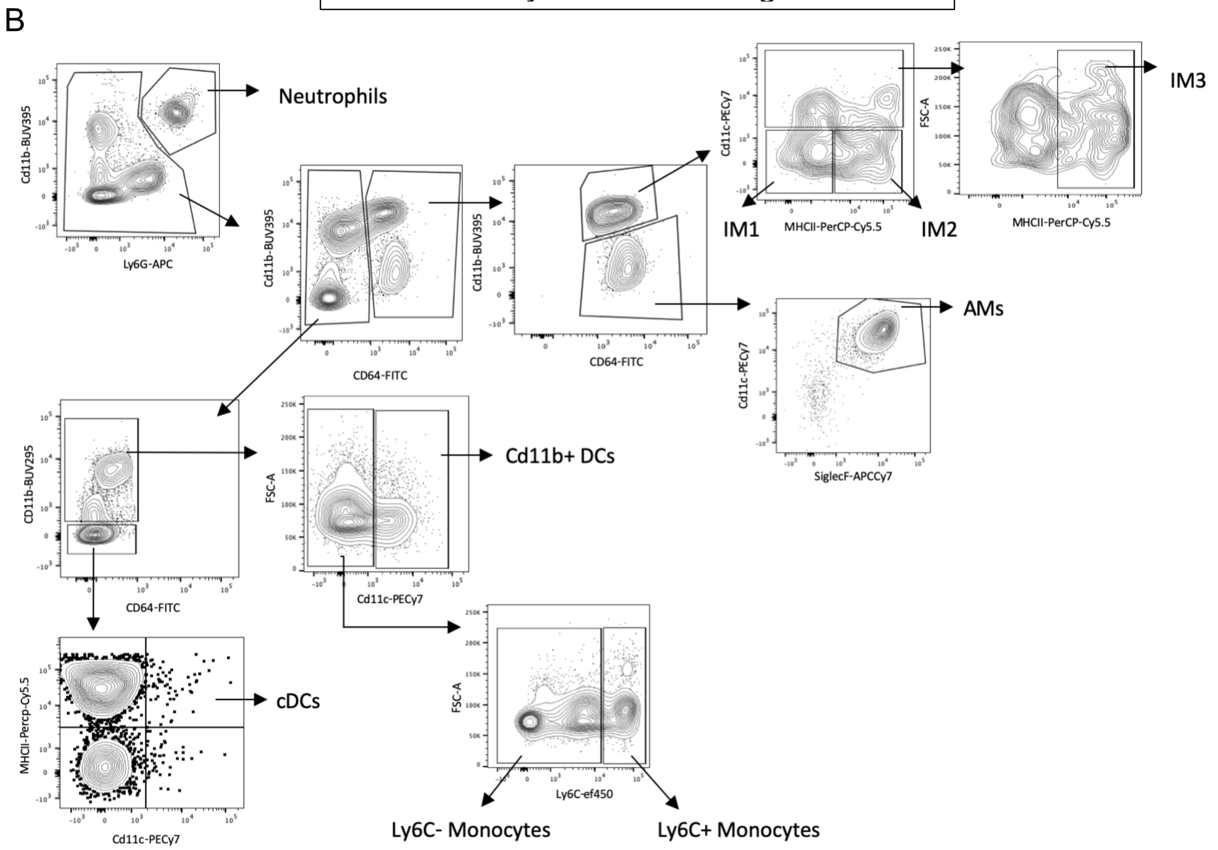

Figure S7: Flow cytometry gating strategies to determine LOX-1 expression on myeloid cell populations. (**A**) To determine LOX-1 expression on monocytes and macrophages based on cellular compartment, flow cytometry was performed on collagenase digested lungs collected from age-matched, C57BL/6 mice infected intratracheally with *E. coli* for 24h. Mice were also given intravenous α-CD45.2-PercpCy5.5 antibody 3 minutes prior to euthanasia to discriminate intravascular from extravascular cells. Using this strategy we can separate alveolar macrophages (pan-CD45+/F4/80+/SiglecF+), intravascular neutrophils (pan-CD45+/iv-CD45+/Ly6G+), intravascular macrophages (pan-CD45+/iv-CD45+/Cd11b+/SiglecF-/F4/80+), intravascular monocytes (pan-CD45+/iv-CD45+/Cd11b+/F4/80-/Ly6C+), extravascular neutrophils (pan-CD45+/iv-CD45-/Ly6G+), extravascular macrophages (pan-CD45+/iv-CD45-/Cd11b+/SiglecF-/F4/80+) and extravascular monocytes (pan-CD45+/iv-CD45-/Cd11b+/F4/80-/Ly6C+). (**B**) Utilizing an expansive panel of myeloid markers, we measured LOX-1 median fluorescence intensity on cells collected from collagenase-digested lungs from age-matched C57BL/6 mice treated intratracheally with saline or *E. coli* for 24h. Using this strategy we were able to determine LOX-1 surface expression on alveolar macrophages (CD64+/CD11b-/CD11c+/SigF+), three subsets of interstitial macrophages (IMs), IM1 (CD64+/CD11b+/CD11c-/MHCII-), IM2 (CD64+/CD11b+/CD11c-/MHCII+), and IM3 (CD64+/CD11b+/CD11c+/MHCII+), CD11b+ dendritic cells (DCs) (CD64-/CD11b+/CD11c+), conventional DCs (CD64-/CD11b-/CD11c+/MHCII+), Ly6C+ monocytes (CD64-/CD11b+/CD11c-/Ly6C+), Ly6C- monocytes (CD64-/CD11b+/CD11c-/Ly6C-) and neutrophils (CD64-/CD11b+/Ly6G+).

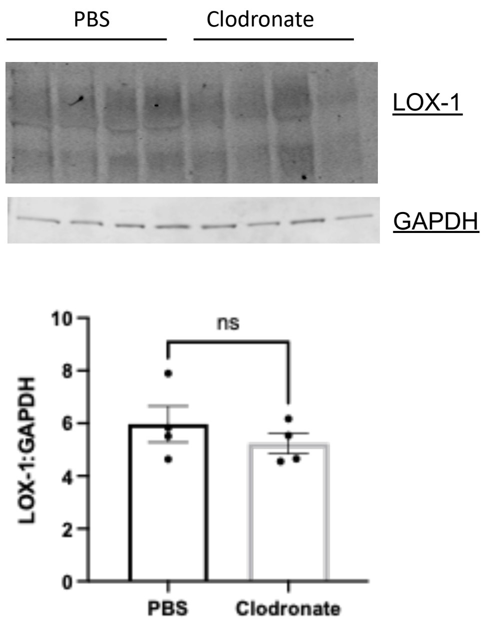

Figure S8: Alveolar macrophage depletion does not affect the inducible pool of total lung LOX-1 during pneumonia. To determine the specific contribution of AMs to LOX-1 induction in whole lungs following infection, C57BL/6 mice were treated intranasally with either PBS or clodronate liposomes, following by intratracheal infection with *E. coli* for 24h. LOX-1 was measured by immunoblot in whole lung homogenates and quantified using densitometry.

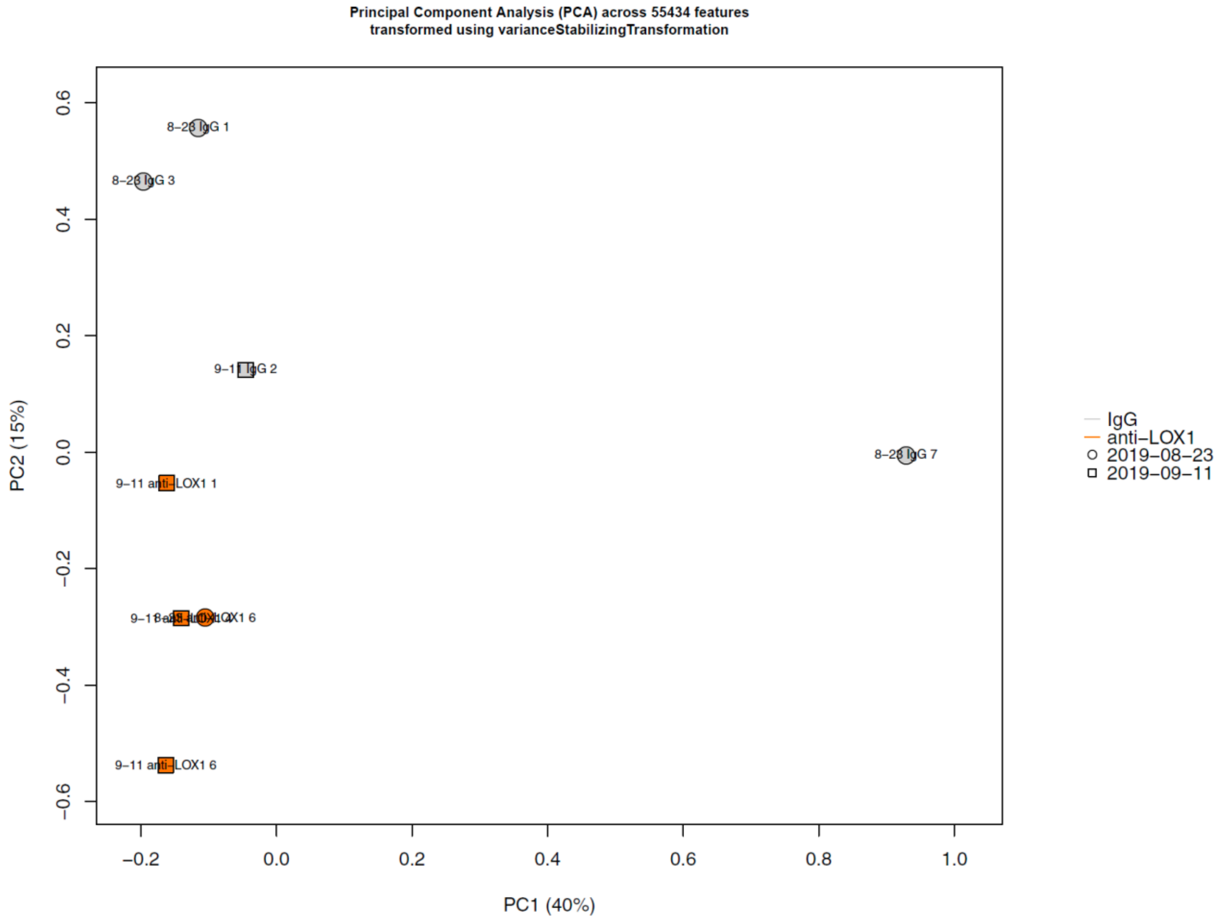

Figure S9: Principal component analysis of alveolar macrophage transcriptional profile from IgG and anti-LOX-1 treated mice during pneumonia. A principal component analysis was performed on the total transcriptional profile of alveolar macrophages isolated from age-matched C57/BL6 mice (n=4 per group) that were intratracheally treated with 10 µg anti-LOX-1 or control IgG and infected with *E. coli.* Gene count data was normalized using the DESeq R package using a variance stabilizing transformation to control for variance and library size. The values for each gene were then *z*-normalized across all samples. Meta-variables arranged across PC1 explains the most variance within samples, followed by PC2.

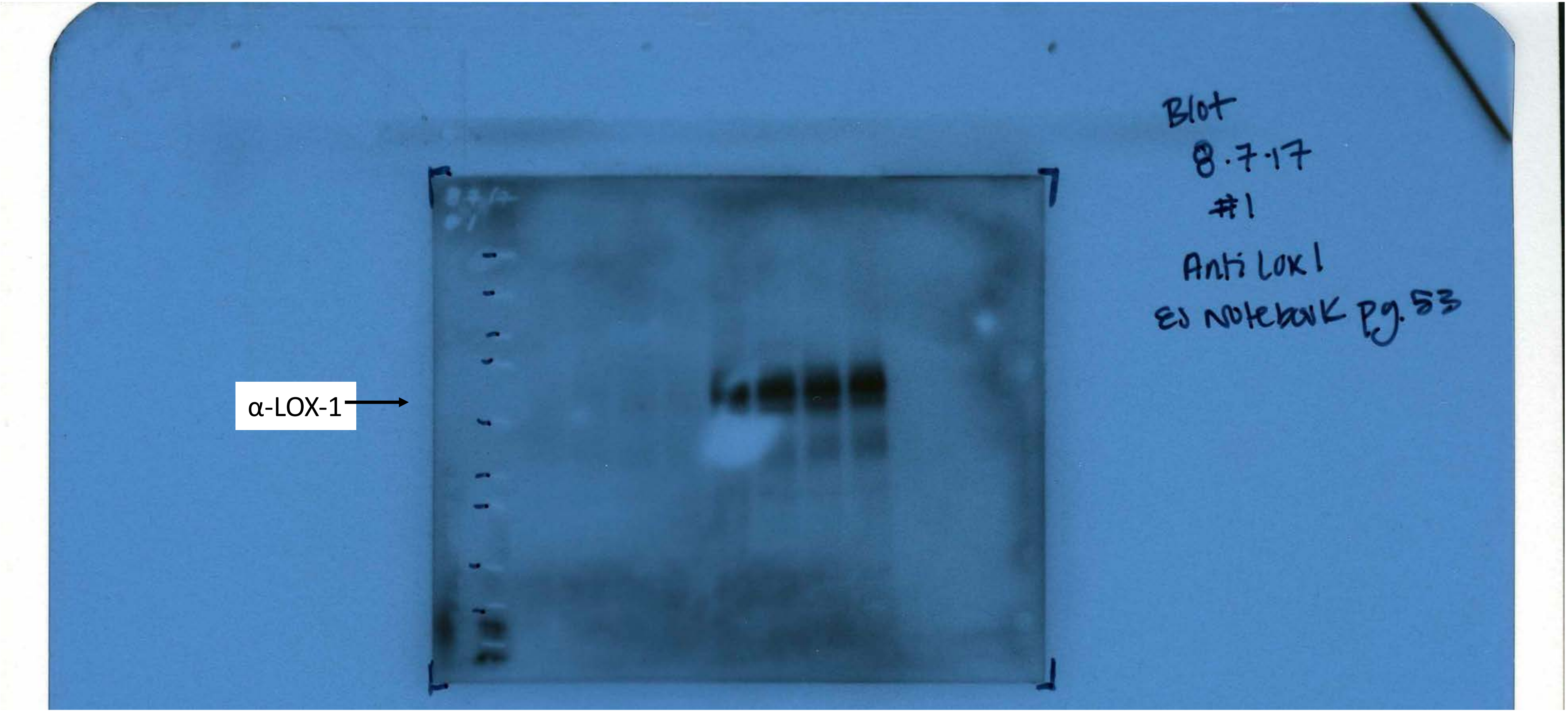

Full unedited gel for Figure 1A
24h saline or *E. coli*

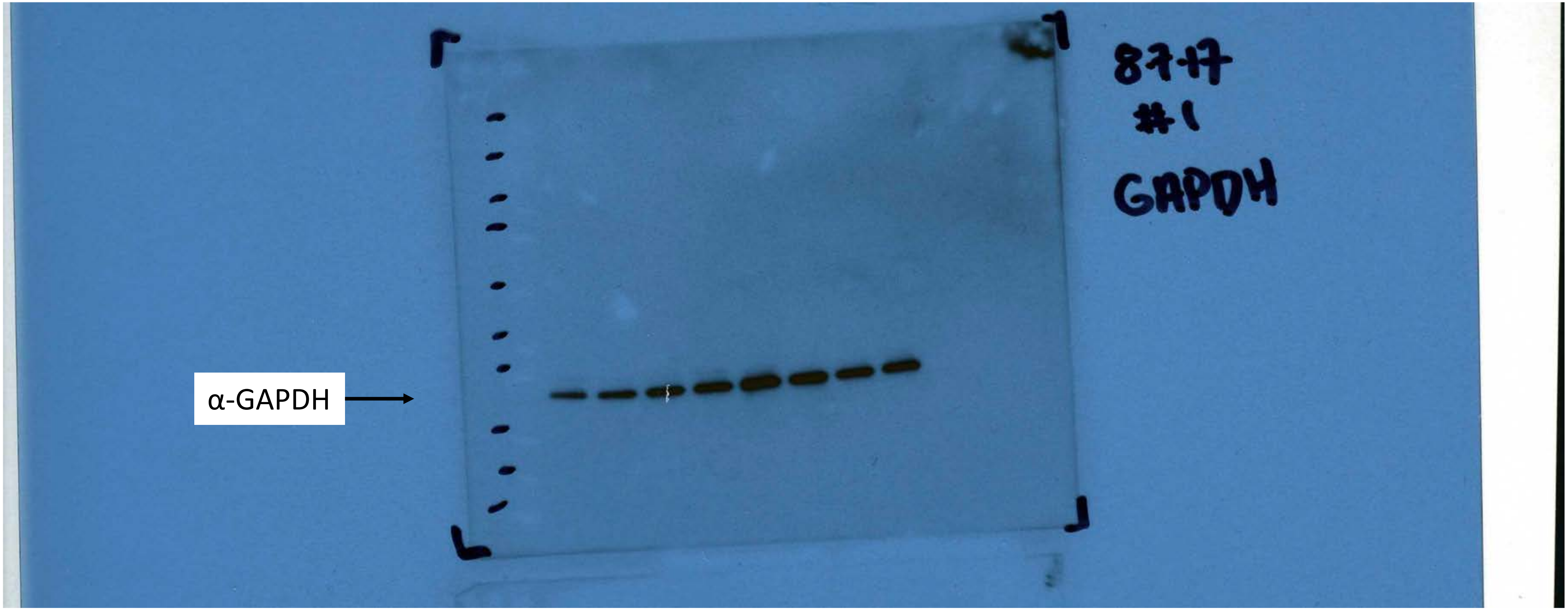

Full unedited gel for Figure 1A
24h saline or *E. coli*

Full unedited gel for Figure 1A

0, 4, 24h *S. pneumoniae*

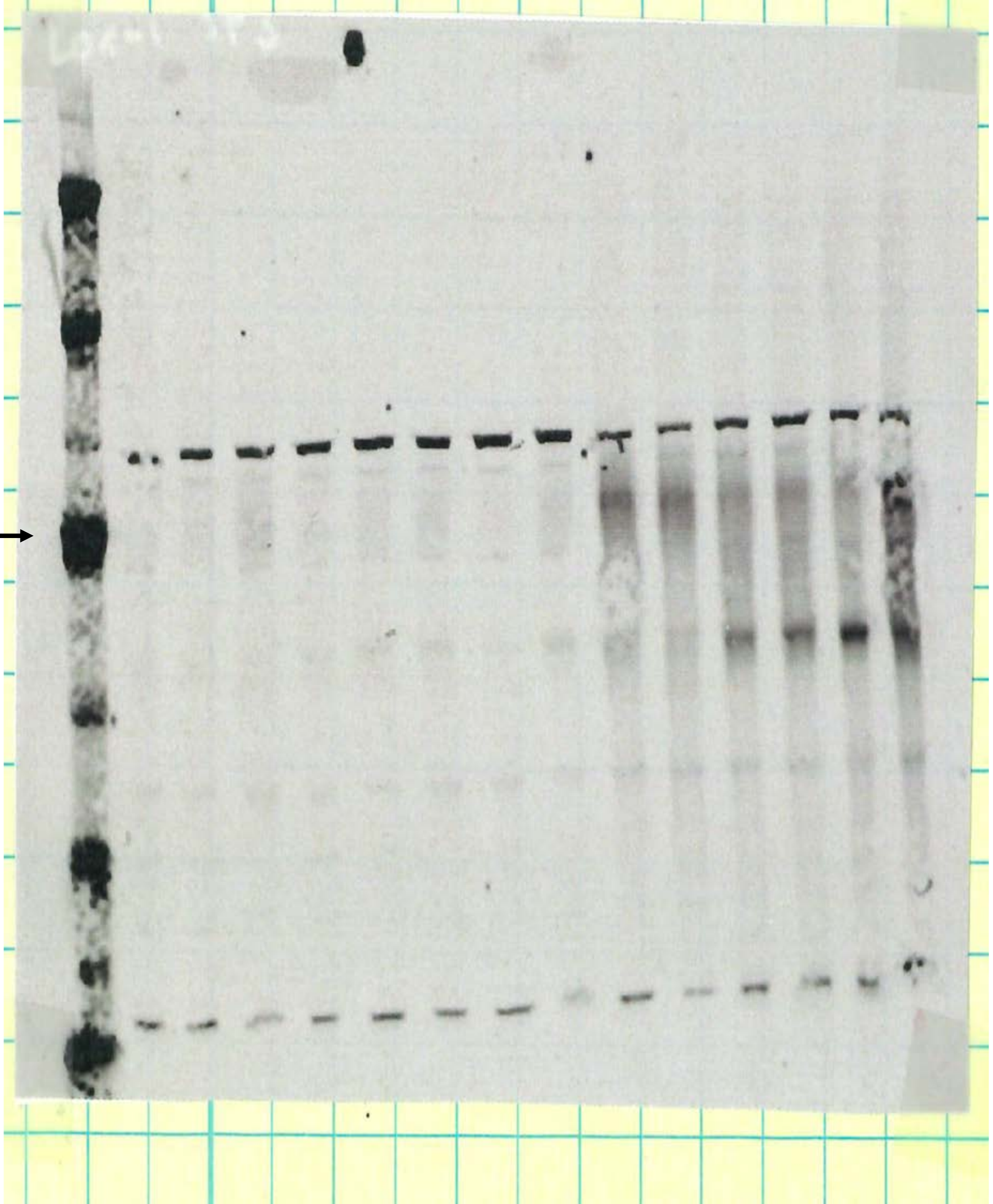

α-LOX-1

Full unedited gel for Figure 1A

0, 4, 24h *S. pneumoniae*

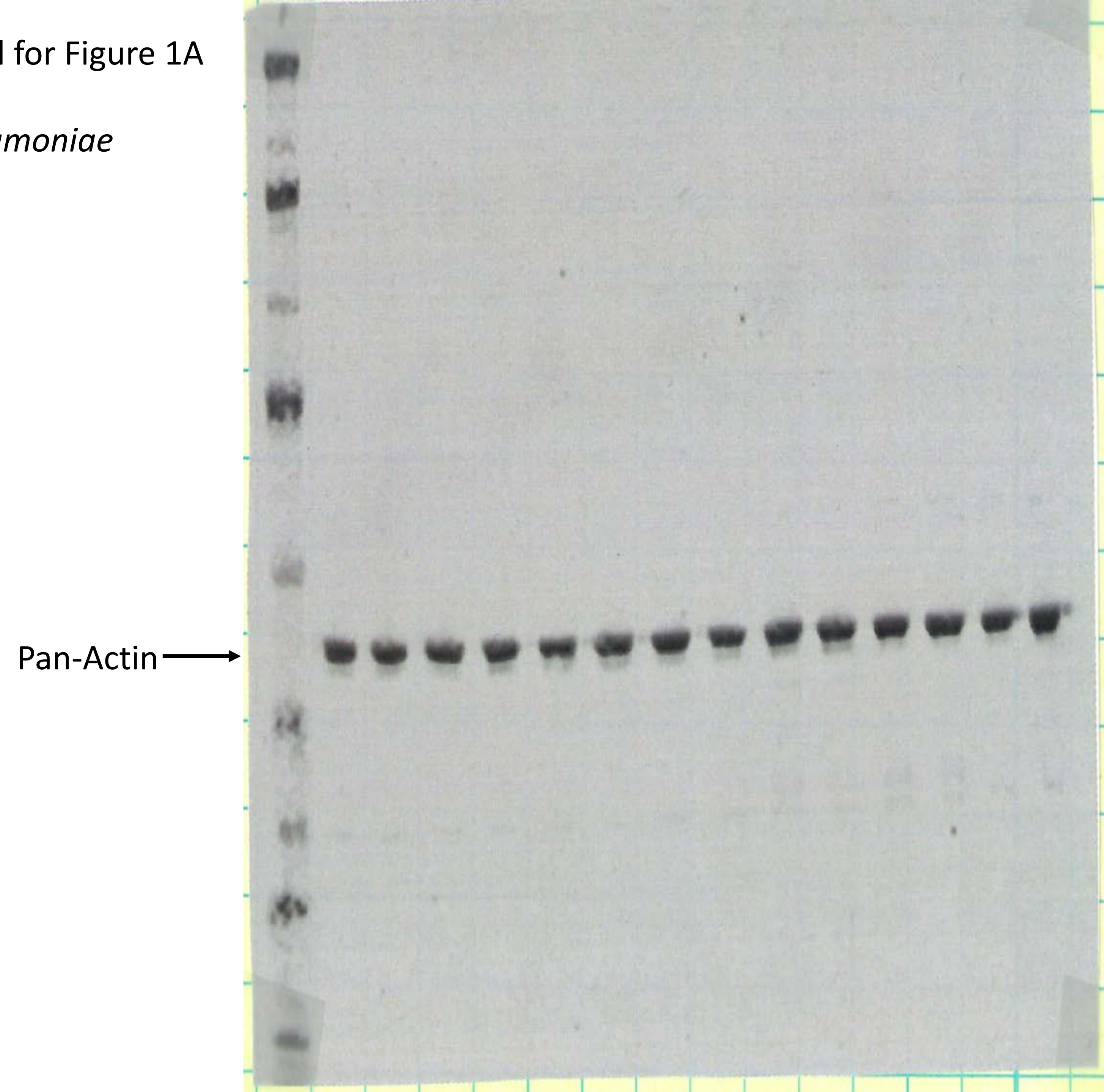

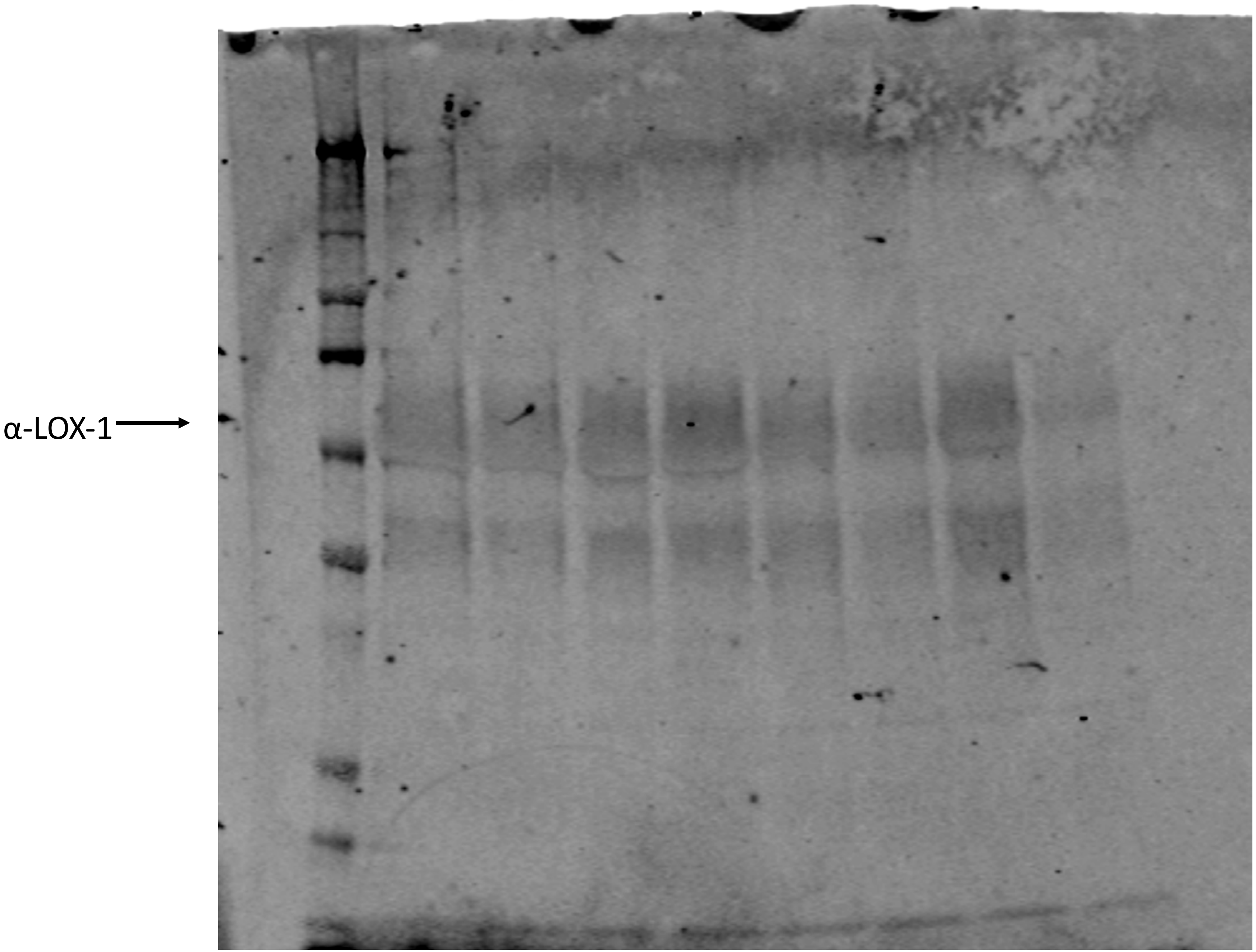

Full unedited gel for Figure S8
24h *E. coli* +/- clodronate

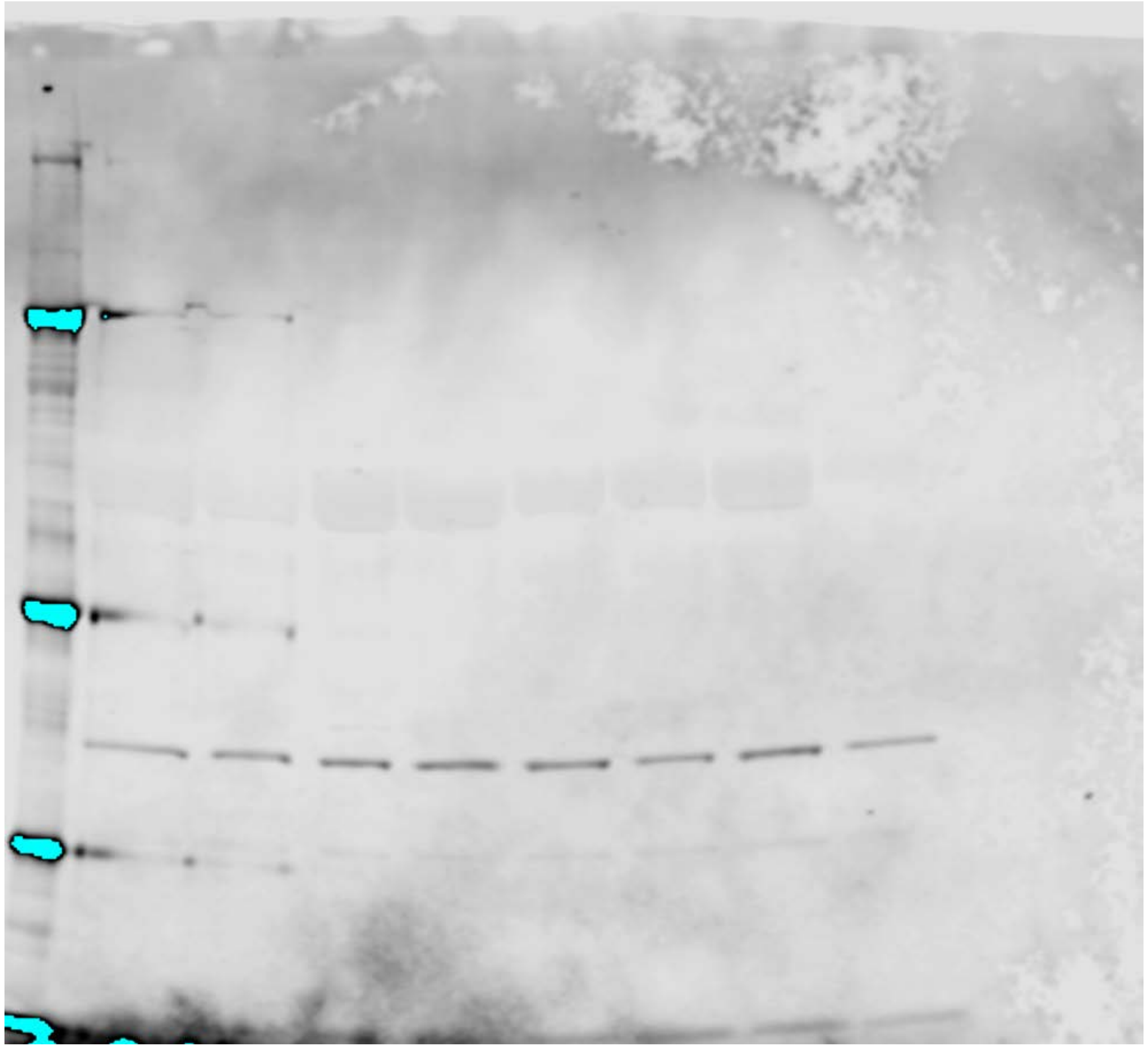

α-GAPDH

Full unedited gel for Figure S8
24h *E. coli* +/- clodronate
